# HFOApp: A MATLAB Graphical User Interface for High-Frequency Oscillation Marking

**DOI:** 10.1523/ENEURO.0509-20.2021

**Published:** 2021-10-08

**Authors:** Guangyu Zhou, Torben Noto, Arjun Sharma, Qiaohan Yang, Karina A. González Otárula, Matthew Tate, Jessica W. Templer, Gregory Lane, Christina Zelano

**Affiliations:** 1 Department of Neurology, Feinberg School of Medicine, Northwestern University, Chicago, Illinois 60611; 2 Department of Neurology, The University of Iowa, Iowa City, Iowa 52242; 3 Department of Neurosurgery, Feinberg School of Medicine, Northwestern University, Chicago, Illinois 60611

**Keywords:** graphical user interface, high-frequency oscillations, MATLAB

## Abstract

Epilepsy affects 3.4 million people in the United States, and, despite the availability of numerous antiepileptic drugs, 36% of patients have uncontrollable seizures, which severely impact quality of life. High-frequency oscillations (HFOs) are a potential biomarker of epileptogenic tissue that could be useful in surgical planning. As a result, research into the efficacy of HFOs as a clinical tool has increased over the last 2 decades. However, detection and identification of these transient rhythms in intracranial electroencephalographic recordings remain time-consuming and challenging. Although automated detection algorithms have been developed, their results are widely inconsistent, reducing reliability. Thus, manual marking of HFOs remains the gold standard, and manual review of automated results is required. However, manual marking and review are time consuming and can still produce variable results because of their subjective nature and the limitations in functionality of existing open-source software. Our goal was to develop a new software with broad application that improves on existing open-source HFO detection applications in usability, speed, and accuracy. Here, we present HFOApp: a free, open-source, easy-to-use MATLAB-based graphical user interface for HFO marking. This toolbox offers a high degree of intuitive and ergonomic usability and integrates interactive automation-assist options with manual marking, significantly reducing the time needed for review and manual marking of recordings, while increasing inter-rater reliability. The toolbox also features simultaneous multichannel detection and marking. HFOApp was designed as an easy-to-use toolbox for clinicians and researchers to quickly and accurately mark, quantify, and characterize HFOs within electrophysiological datasets.

## Visual Abstract

**Figure:**
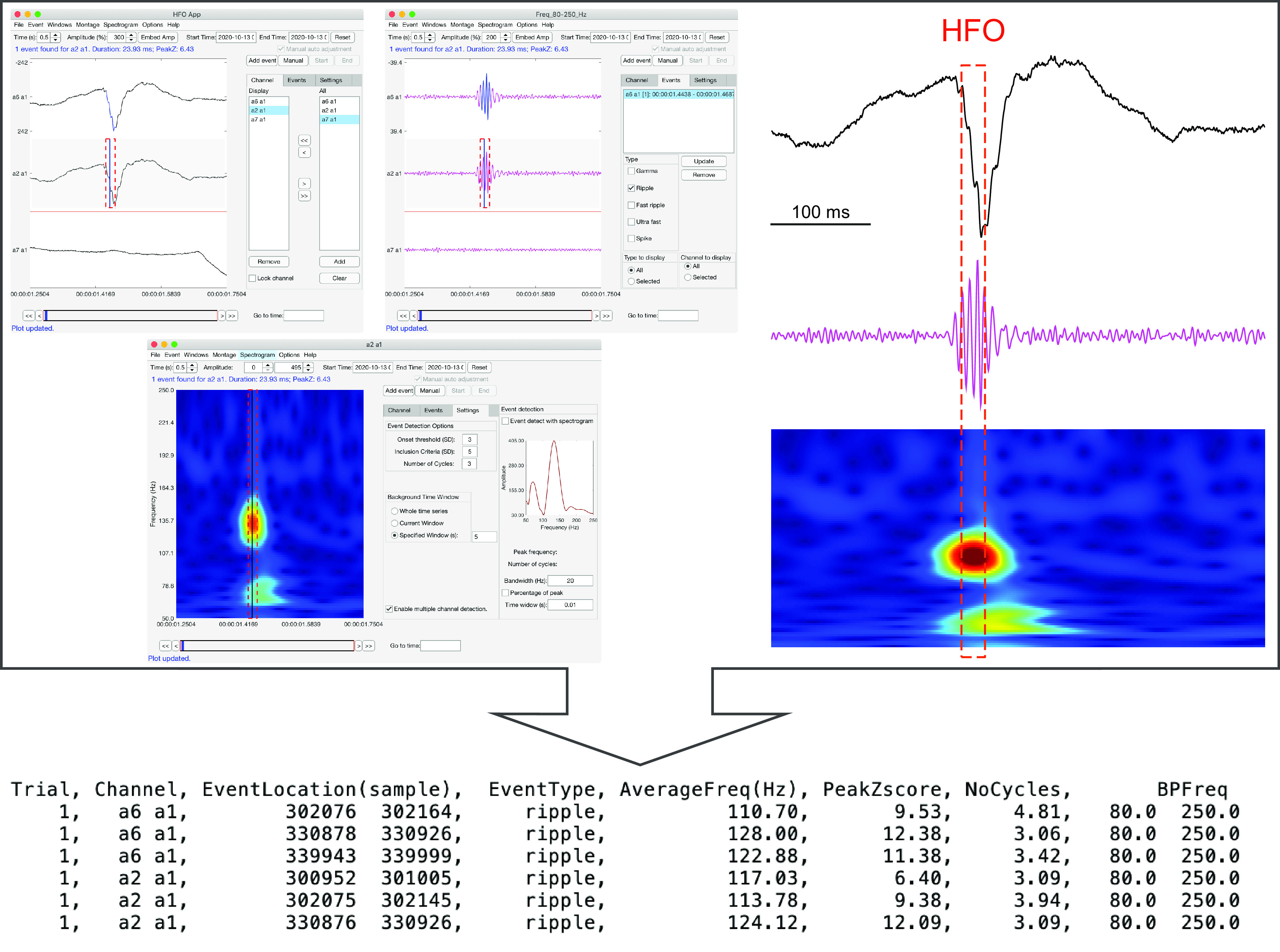

## Significance Statement

We introduce a MATLAB-based graphical user interface designed to facilitate visual marking of high-frequency oscillations in electrophysiological data by prioritizing usability, speed, and accuracy. It allows clinicians and researchers to quickly and easily visualize and mark multiple channels of raw and bandpass-filtered data simultaneously, either in the same window or in separate windows, facilitating fast and accurate discrimination between real high-frequency oscillations and spike artifacts. The implementation of both automatic detection and interactive automation-assist options significantly speeds up visual marking of high-frequency oscillations. The simple data structure used by the toolbox also increases its ease of use. These features make it a useful toolbox that is valuable in the field.

## Introduction

Epilepsy is one of the most common neurologic disorders, affecting 3.4 million individuals in the United States (Zack and Kobau, 2017). Despite the availability of numerous antiepileptic drugs, ∼36% of epilepsy patients live with uncontrolled seizures, which is debilitating and causes increased healthcare resource use. With average hospital stays of 3.6 d, the aggregate hospital costs for epilepsy totaled ∼$2.5 billion in 2014 [Healthcare Cost and Utilization Project, 2014 National Data (https://www.hcup-us.ahrq.gov/)].

For a subset of patients with uncontrolled seizures, surgical removal of epileptic brain tissue has been demonstrated to be an effective treatment. This surgery typically requires extensive presurgical evaluation to define the resection target. High-frequency oscillations (HFOs) hold promise as a new biomarker for epileptogenic tissue (Engel et al., 2009; Zijlmans et al., 2012; Roehri et al., 2018), particularly for patients undergoing presurgical evaluations. Identification and localization of HFOs during presurgical evaluations can help to define resection targets by pinpointing pathologic tissue, the removal of which has been shown to improve surgical outcomes, reducing future seizure activity (Worrell et al., 2004; Jacobs et al., 2010; Blanco et al., 2011; Fujiwara et al., 2012; Zijlmans et al., 2012; Höller et al., 2015; Gliske et al., 2016; Frauscher et al., 2017, 2018; Velmurugan et al., 2019).

Automated detection of HFOs remains challenging, as it produces inconsistent results across methods, though several automatic detectors have been developed (Crépon et al., 2010; Zelmann et al., 2010, 2012; Cimbalnik et al., 2018; Donos et al., 2020; Lachner-Piza et al., 2020; Lai et al., 2020; for review, see Remakanthakurup Sindhu et al., 2020). One significant challenge that remains for automatic detectors is distinguishing HFOs from artifactual spikes as well as epileptiform EEG spikes, because their spectrograms overlap. Therefore, to accurately identify HFOs, it is important to examine not only the bandpass-filtered data, but also the raw unfiltered data. Thus, visual identification with manual marking of HFOs is still widely accepted as the gold standard (Zelmann et al., 2010).

To best visually identify HFOs, the raw data are bandpass filtered into high-frequency bands, typically 80–250 Hz for ripples and 250–500 Hz for fast ripples. Then, the bandpass-filtered signal and raw data are reviewed together. Automated detection of HFOs can assist in this process, but manual review of automated results is still necessary. Therefore, there is a need for a software toolbox that can take user-inputted intracranial electroencephalographic (iEEG) data, display the raw data of each channel along with bandpass-filtered and spectrogram data, allow for quick and easy manual marking of HFOs, and output detailed parameters of each HFO event. While many researchers create custom MATLAB scripts to assist in specific research projects involving manual marking of HFOs, there are very few universal, open-source HFO detection applications (Navarrete et al., 2016). Here, we developed HFOApp, a broadly usable, open-source HFO detection toolbox that incorporates critical features to improve on existing toolboxes to increase usability, improve speed, and decrease variability between users, thus increasing accuracy.

## Materials and Methods

### Summary of improvements over existing open-source HFO applications

HFOApp allows simultaneous marking of multiple channels, in contrast to other HFO applications. In HFOApp, the user can do this by simply clicking between channels: a click on any channel in any open display window will allow marking on that channel. In addition, within channels, HFOs can be marked on the raw data or on any filtered data or spectrogram window. The user can choose to simultaneously display and mark as many data channels and as many bandpass-filtered time series windows as needed.

#### Flexible display of data

HFOApp allows visualization of raw, filtered and spectrogram data in separate, but synchronized, windows, which allows the user flexibility in positioning different data display types on their monitor during marking, according to their needs. HFOApp also allows the display of filtered data below the raw data in the same window.

#### Usability

We designed HFOApp with the following three main usability principles in mind: using the software should be (1) intuitive, (2) comprehensible, and (3) familiar. The software should not require extensive instruction to navigate, because it uses controls based on software typically in use in clinics and research laboratories. Typical users will be acquainted with the controls without training, the controls will be where users expect them to be, and they will operate in a sensible way. Specifically, the use of drop-down menus, list boxes, and keyboard shortcuts are all designed for ease of use. In addition, HFOApp automatically saves results as you go, minimizing accidental loss of data. Because of this, manual and automation-assisted marking of events is intuitive and fast.

#### Interactive automation-assisted manual marking on raw and filtered time series

HFOApp allows interactive automation-assisted manual marking. During a typical manual marking session, a user will navigate through the data in time windows of ≤1 s, visually examining the data in each time window for HFOs. If an HFO is found, the user then marks its start and end manually and adds the event to the results file. HFOApp improves on this process by offering the following optional automated assistance feature: as users advance through data, they have the option of using an automated detector to identify events within the current time window, across all displayed channels, by simply pressing “d” (for “detect”). When “d” is pressed, the HFOApp will outline any potential HFOs detected in that time window based on user-set parameters, including baseline length, cycle count, amplitude above baseline, and onset threshold. The user can then easily interact with the outlined events: they press “a” (for “add”) if they agree that all detected events in that time window should be marked, and all events will be marked and added to the Events List, including events found across multiple channels. If the user disagrees with any event, right clicking on the event will deselect it, removing it from the display of potential events. This automation-assisted detection greatly speeds identification, selection, and deselection of events, allowing manual marking with ease. Using keyboard shortcuts for this feature eases the user’s ergonomic burden: users can simply press “f” (for “forward”) to move to the next 1 s window, press “d” to view and assess potential events, make any needed modifications using the mouse, then press “a” to add all marked events to the Events List. This series of button pressing can be repeated through the marking procedure, making it extremely fast and streamlined. In addition, the user can simultaneously perform manual marking of events not detected by the automated detector. Note that as manual marking of events is performed, it is also assisted by optional, user-defined automated threshold detection, increasing accuracy and consistency across raters.

#### Interactive automation-assisted manual marking on spectrogram data

HFOApp also offers the option of automatic detection within a narrow frequency band with a mouse click in the synchronized spectrogram window. This automated frequency-band detection function is quick and easy to use during manual marking, and significantly enhances the detection of events that occur over a narrow frequency band. To use this function, the user clicks on an event in the spectrogram display window, and an event-specific bandpass-filtering function is executed, recalculating user-set event parameters within a narrow bandwidth, thus optimizing the signal-to-noise ratio for that event at the selected (clicked) frequency. For example, a user marking the bandpass-filtered signal might come across a complex event that may be an HFO, but the automatic-assist detector does not detect it due to EEG artifacts across the broadband signal. In cases like this, the user can make use of the automatic frequency-band detection feature in the spectrogram window. In this synchronized window, frequency-specific components of the event are easily visualized, with any frequency-specific peaks shown in both the spectrogram itself and in the frequency–amplitude graph in the window sidebar, which displays information for a small time window around the cursor location. The user can simply click on the peaks in the spectrogram window (easily seen as bright color differences), which will prompt HFOApp to automatically execute a bandpass filter within a user-set range and recalculate automated HFO detection within that narrow frequency range, encompassing only the cluster that was clicked. If an HFO is detected, it will be automatically outlined on all synchronized display windows, including the spectrogram, the raw data, and any bandpass-filter display windows; the user can add it to the Events List by pressing “a”.

#### App designer

In keeping with the MathWorks recommendations for the development of Apps within MATLAB, we used App Designer instead of GUIDE. App Designer is the MATLAB replacement for GUIDE [https://www.mathworks.com/products/matlab/app-designer/comparing-guide-and-app-designer.html (accessed on July 12, 2021)], and it offers a vastly expanded set of features and functions, making it more versatile, easier to code, and easier for maintenance and customization. Notably, MathWorks has announced that it will offer ongoing support for and updates to App Designer, while also issuing notification that GUIDE will be removed from future releases of MATLAB. Therefore, GUIDE is, both in terms of features and support, obsolete. As prior MATLAB-based HFO toolboxes were based on GUIDE, the use of App Designer for HFOApp is another advantage over those applications.

This dynamic combination of ease of use, comprehensibility of design, flexibility of implementation, optional automation assist for on-the-fly use at multiple levels of manual marking, and an architecture built on an active, supported platform makes HFOApp a versatile and powerful HFO marking tool, with significant improvements over existing applications.

### Overview of HFOApp

**Figure 1.**
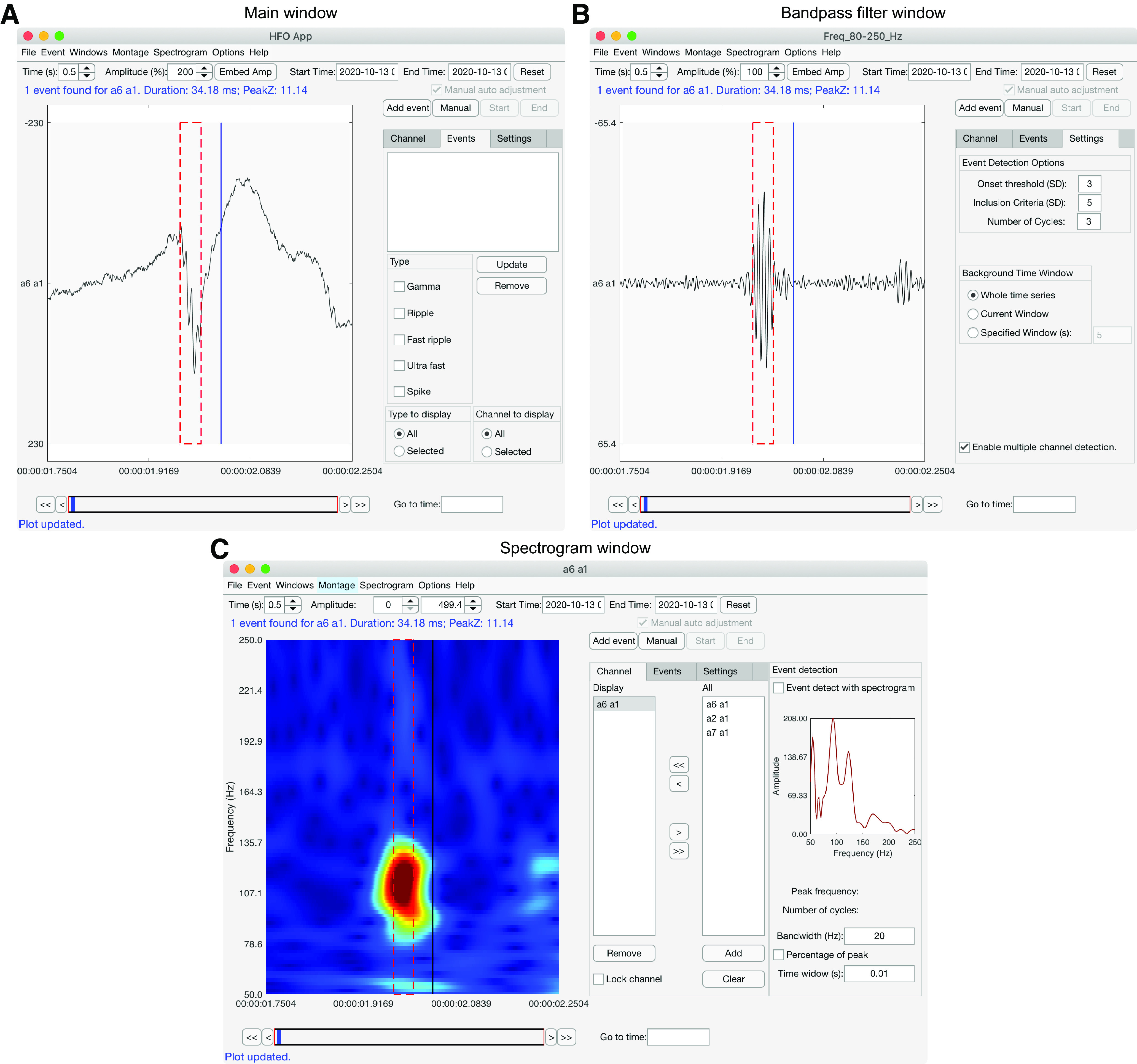
Example usage and output of HFOApp. ***A–C***, To visually detect HFOs in our test dataset, we opened the following three windows: a main window that shows raw data (***A***); a bandpass-filtered window (80–250 Hz for ripple detection; ***B***); and a spectrogram window for the first channel (***C***). The cursor is indicated as a blue (***A***, ***B***) or black (***C***) vertical line. The red dashed rectangle indicates the boundaries of a detected HFO. A summary of the characteristics of the detected event is shown above the plot in blue font.

HFOApp offers automated and manual HFO detection and marking options with a high degree of flexibility and user control. To use HFOApp, the user simply imports iEEG data and can then choose to run any of five preloaded automated detectors or add another automatic detector by following instructions in the manual. For manual marking, the user can first set the data display according to their preference. HFOApp allows display of raw iEEG data, bandpass-filtered data, and spectrograms embedded either into the same window or into two or more separated, but automatically synchronized, windows (Fig. 1*A–C*). This flexibility is very useful, as individual preferences may differ depending on how many channels are being reviewed, the size of the monitor being used, and other visual factors. Additionally, the user can generate as many bandpass-filtered data displays as needed, each with different user-defined frequency limits. To optimize the display for manual marking, the user can set viewing parameters, such as the length of time displayed in the window, the amplitude of the data, the color of the displayed time series. The user can then scroll through the data, marking the start and end locations of HFO events manually, with ergonomically efficient controls, such as using the mouse to position the cursor and using keyboard shortcuts to scroll through the data and to mark events. Events can be easily added to or removed from the Events List. Controls for the identification, marking, and management of events are intuitive and comprehensible.

HFOApp allows for simultaneous review and marking of multiple channels. The user can display as many channels and bandpass-filter windows as needed, reviewing and marking within any displayed window. All displayed data windows are synchronized by default. The toolbox was designed such that the user can easily switch between channels by simply clicking on any time series window.

To assist with manual review and marking, we introduce an interactive, automatic Hilbert detector assist feature that can be used on the fly within the graphical user interface (GUI). This feature allows users who are manually reviewing data to simultaneously use automatic detection to identify all events in all channels in the current time window. As the user manually scrolls through the data, they can deploy the automatic detector on their current time window with a button press, which prompts HFOApp to outline any HFOs that were detected by the automated algorithm. Identified HFOs are then easily managed with mouse and keyboard controls. In addition, when the user manually marks an HFO, they can use the automated assist to define onset thresholds for that event.

An additional strength of HFOApp is the introduction of a dynamic, automated frequency-band determination for filtering based on the peak frequency of the spectrogram, designed to increase the sensitivity of the Hilbert detector. The first step of most automatic detectors includes bandpass filtering the data into distinct, but broad, frequency bands. However, broadband display can make discrimination between artifactual and real HFOs difficult in cases where frequency components of both are present within the broad band. HFOs may be apparent only in a narrow band (Gliske et al., 2017), and, therefore, detection power could potentially be weakened by using broadband filtering. To address this problem, HFOApp allows users to simply click on hotspots in the spectrogram to initiate automated narrow-band detection, based on user-defined parameters.

### Using HFOApp

HFOApp requires a MATLAB version from 2019 on (2019a&b, 2020a&b, and 2021a) and will be kept up-to-date with future MATLAB releases. In typical usage, once iEEG data are loaded into HFOApp (see the section Data structure), the raw data are displayed in a time series data window (Fig. 1*A*). One or more bandpass-filtered time series can then be displayed, embedded either in the original data window as a new row below the raw data or in a new window (Fig. 1*B*). A spectrogram window can also be generated as a separate window (Fig. 1*C*). All windows are automatically synchronized; as the user scrolls through the data in any window, all windows move in tandem.

The data can be analyzed for marking HFOs in the following four ways: manual, manual with interactive automation-assisted thresholding, manual with interactive automation-assisted detection, and fully automatic. Within all of these methods, HFOApp allows for simultaneous analysis and marking of multiple data channels, broad flexibility of display, and a high degree of usability. For the three manual marking methods, the user can display and mark as many channels, bandpass filter windows, and spectrogram windows as needed. These windows are user-generated using plain-language, intuitive controls on popup windows prompted by drop-down menu selections.

### Four marking methods of HFOApp

#### Manual marking of HFOs

To mark HFOs without using any automated features, the user sets the fully manual mode by clicking on the “Manual” button, and deselecting the “Manual auto adjustment” box, both of which are on the top right of the display window (Fig. 1*A*,*B*). The user then scrolls though the data using either clickable controls on the GUI or keyboard shortcuts. At any time, the user can click on the time series display in any bandpass-filtered data window and the cursor location will appear as a vertical line (Fig. 1*A*), with the display showing the *z* score of the envelope of the bandpass-filtered data at the cursor location. The user can use this information to help determine whether an event is an HFO and, if so, where the start and end points are located. When an HFO is identified, the user simply positions the cursor at the start of the event and presses “s” (for “start”), then positions the cursor at the end of the event and presses “e” (for “end”). The event will then be outlined with a red dashed line, using the marked start and end points, on all windows displaying that channel, including the raw data. Information about the selected event will be immediately displayed, including the peak *z* score, the duration, and the number of cycles. If the user wants to change the event, they can do so by resetting the start or end points as above. If the user is satisfied with the marking, they can add the event to the Events List by pressing “a” on the keyboard. If the event was added by mistake, the user can select the event in the Events List and press the “Remove” button below the Events List to delete it. The results will be automatically updated whenever an event is added or removed from the Events List if automatic saving was enabled.

#### Manual marking of HFOs with interactive automation-assisted thresholding

To assist with manual marking, HFOApp features interactive automated thresholding to calculate the start and end points of events, to speed marking and improve accuracy. To use this feature, the user sets manual mode by clicking on the “Manual” button at the top right of the display window (Fig. 1*A*,*B*), and confirms that the “Manual auto adjustment” box just above the “Manual” button is selected (it is selected by default). Then, when the user follows the steps for marking an event (detailed above), the manually selected start and end points are replaced using automatically generated thresholds. This provides the ability to set a precise, consistent, and accurate duration of every event, based on user-selected parameters (see next section). In practice, this feature allows the user to approximately set the start and end points of HFO events, and then rely on the HFOApp automation to adjust to precisely accurate points, greatly speeding marking while increasing accuracy. This feature is interactive because the user has fine control over the settings used by the automation and can deploy it on-the-fly by selecting or deselecting the option while marking. The operations of the thresholding function are detailed below.

#### Manual marking of HFOs with interactive automation-assisted detection

**Figure 2.**
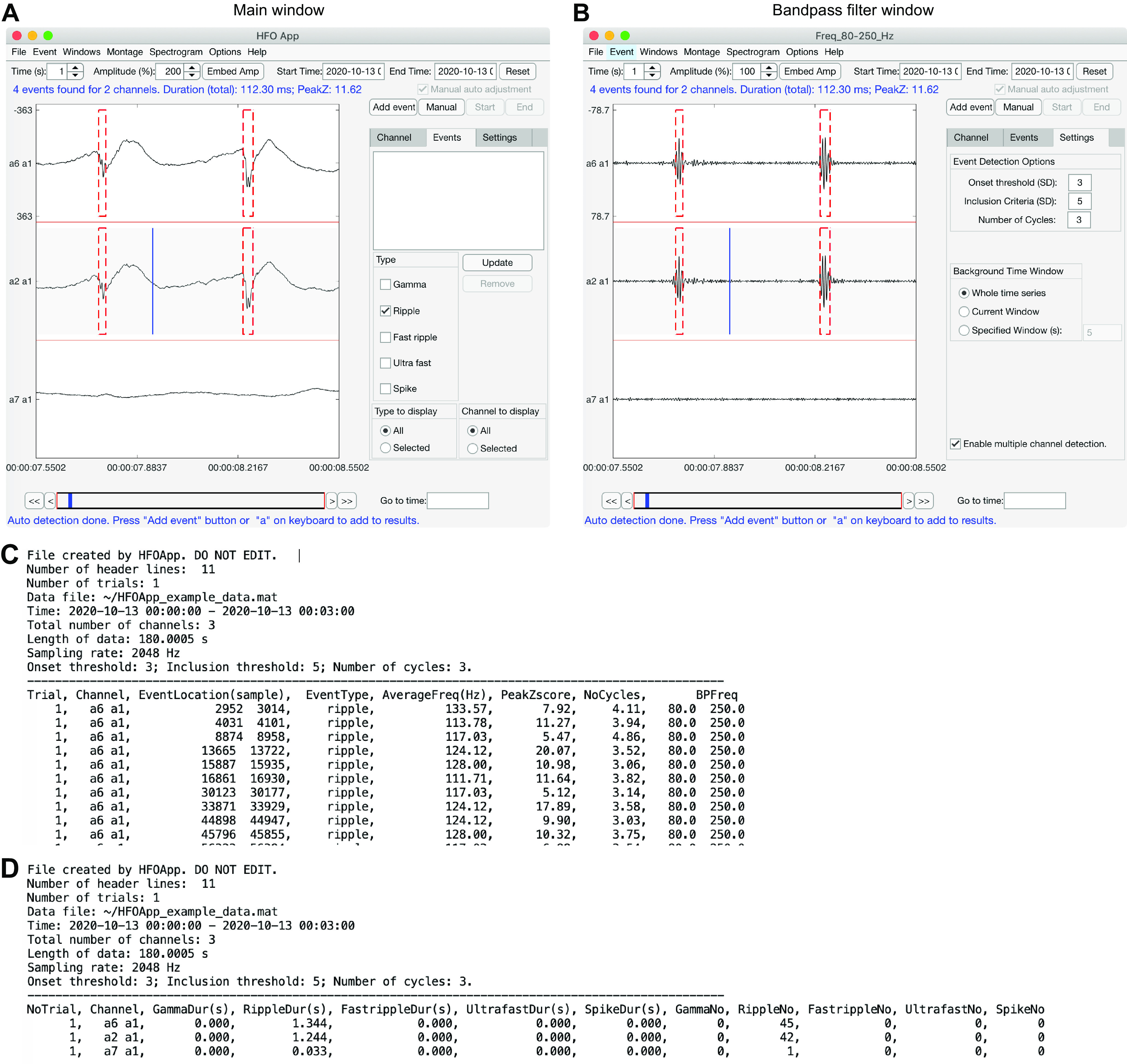
Multiple-channel HFO detection. When the “Enable multiple channel detection” option is enabled, all events for all channels in the current time widow can be detected by pressing “d” on the keyboard. ***A***, ***B***, Illustration of the automatically synchronized main (raw) and bandpass-filtered windows of the toolbox. The blue vertical line indicates the cursor location, and the red dashed rectangle indicates detected HFOs. ***C***, Example of formatted output of HFOApp. Each row shows detailed information about each event. The first 10 events are shown. ***D***, A summary of events for each channel.

To make manual marking even faster and more accurate, HFOApp features manual marking with interactive automation-assisted detection. This powerful automated detection tool can be quickly deployed on-the-fly during manual marking with both keyboard shortcuts and mouse clicks, while providing the user with fine control over parameters used by the automation. HFOApp starts in this setting by default; no action is needed to set this mode. As the user scrolls through the data, they can trigger automated detection of HFOs within any displayed bandpass-filtered time series in the following two ways: by simply clicking anywhere on a bandpass-filtered time series (to detect events within that channel) or by pressing “d” on the keyboard (to detect events across all channels). When the user clicks in a data window, the cursor location appears as a vertical line (Fig. 1*A*) and the *z* score of the time series is thresholded according to the user-set HFO-onset threshold [Fig. 1*B*, Onset threshold (SD)]. Any clusters that survive this initial threshold will be identified within a contained search range (see section Implementation of the Hilbert detector). A cluster will be considered an HFO event if it meets the following two criteria: (1) the maximal *z* score within the cluster meets the inclusion threshold, which is typically larger than the onset threshold [Fig. 1*B*, Inclusion threshold (SD)]; and (2) the number of oscillatory cycles is greater than a certain threshold (Fig. 1*B*, Number of cycles). Figure 1 shows an example of HFO detection using the toolbox. In this example, we display the raw data (Fig. 1*A*) and the bandpass-filtered data (Fig. 1*B*) in two distinct windows, which is useful when analyzing a large number of channels. Using the keyboard shortcut “d” to run automated detection will perform the above functions over all displayed channels and windows (Fig. 2*A*,*B*). Once the multiple-channel detection option is enabled by checking the “Enable multiple channel detection” box (Fig. 2*B*), the user can press “d” on the keyboard to run the automatic HFO detection as above on all channels in the current time window. Detected events that survive inclusion criteria are outlined with a red dashed line (Fig. 2*A*,*B*). Any individual event can be deselected with a right mouse click. Then all selected events can be added to the Events List by pressing “a”. This feature is interactive because it can be deployed on-the-fly across or within channels while manually marking, and because the user has fine control over the detector’s inclusion criteria, including onset threshold, number of cycles, and peak *z* value, as well as the definition of the background. In practice, the user can press “f” (for “Forward”) to scroll to the next time window of data across all synchronized display windows, press “d” to trigger auto detection, press “a” to add detected events to the list, and press “f” to move on to the next window, repeating this sequence while moving quickly through the data. If the user visually identifies an event missed by the automation, they can quickly add it by manually selecting it with automated thresholding, as described in the previous section. This operating mode of HFOApp allows very fast, easy, and accurate visual marking of HFOs.

#### Manual marking of HFOs with interactive automation-assisted detection in the spectrogram

HFOApp also features on-the-fly automatic detection in the spectrogram window. This automated function requires only a single click on the spectrogram, making it quick and easy to use during manual marking, and significantly enhancing detection of events which occur over a narrow frequency band, especially when the broad band is noisy. For example, an HFO may not be apparent in the broad bandpass-filtered time series because the frequency components of both the HFO and artifact overlap. In these cases, reviewers often turn to the spectrogram to look for HFOs in the colormap, where frequency-specific components of the event are easily visualized by color. To make this task faster and more accurate, HFOApp offers an interactive automation-assist feature in the spectrogram window to detect and mark HFOs with a mouse click. This feature executes an automated detector over a narrow frequency and time around any location clicked on the spectrogram colormap. If an HFO event is detected, it is outlined with a dashed red line in all data windows, including the spectrogram, the bandpass-filtered data, and the raw data. The event can then be managed as above. To use this feature, the user selects the “Event detect with spectrogram” box in the “Event detection” section on the right side of the spectrogram window (Fig. 1*C*). The detector determines the peak frequency within a narrow time window (user set in the “Time window” box) around the clicked point, and uses a narrow bandwidth around the peak frequency [user-set in “Bandwidth” box (or can be set as a percentage of the peak by selecting the “Percentage of peak” box)]. If an HFO is detected, the duration, number of cycles, and peak *z* score are displayed, and the event is outlined. This function is interactive because the key parameters can be adjusted during use, allowing for a high degree of flexibility so that the user can quickly adjust to the particular character of the data being analyzed.

### Fully automatic detection

To use the fully automated HFO detectors in HFOApp, the user first selects *Automated HFO detection* from the drop-down menu. This will prompt a popup window labeled *HFO Auto Detectors.* Within this window, the user can choose from five different automatic detectors, including both the HFOApp Hilbert detector (described below) and the RippleLab Hilbert detector (Crépon et al., 2010; Navarrete et al., 2016), the Short Time Energy detector (Staba et al., 2002), the Short Line Length detector (Gardner et al., 2007), and the Montreal Neurologic Institute detector (Zelmann et al., 2012). Once a detector is selected, the options of that detector will populate the window, and the user can set the parameters. Then the user simply clicks the “Run” button. The automated detection will run on all channels loaded in the Display List. Once the automation is finished, the main window will display the message “Automated detection done”, along with the number of new events detected. All new events will be displayed in the Events List. Events can then be manually reviewed by clicking on any event in the list, or by using the arrow keys on the keyboard to scroll through the list. In both cases, as each event is highlighted in the list, it is shown in all display windows (raw and filtered).

### Implementation of additional automated detectors

HFOApp includes five different automated HFO detectors, as described above. In addition, users can easily add HFO detectors to HFOApp, and a detailed explanation is provided in the user manual. In brief, we designed a function (*HFOAutoDetect.m*) that takes a data matrix, a sampling rate, and a configuration data structure as input, and the function is independent from the graphical user interface itself. To configure the input arguments that are required by the new detector, the user simply adds the components to a graphical user interface file (*ConfigAutoDetector.mlapp*).

### Development of the toolbox

The graphical user interface toolbox was developed using App Designer in MATLAB (version R2020b; RRID:SCR_001622). The source code and source data are freely available at https://github.com/zelanolab/hfoapp.git and also as the Extended Data 1.

10.1523/ENEURO.0509-20.2021.ed1 Extended Data 1 HFO App Package V1. Download Extended Data 1, ZIP file.

### Data structure

A detailed description of the data structure for this toolbox is provided in the user manual. Here, we will provide a brief description of the data structure. To begin a session, the raw data are first loaded and preprocessed into the proper format for the graphical user interface. Loading of the data can also be implemented separately from the graphical user interface using the MATLAB function *HFOLoadData.m*, adapted from *fieldtrip* (Oostenveld et al., 2011; RRID:SCR_004849). This allows loading of Micromed and European Data Format files and can be easily modified to support the user’s own data format. The resulting data are formatted into a MATLAB data structure containing the following fields: (1) *mat*, which is a two-dimensional data matrix of channel × sample; (2) *labels*, which is a cell array of channel labels; (3) *srate*, which is the sampling rate; (4) *start_clock*, which is the clock time (the type in format of *yyyy-MM-dd HH:mm:ss* in *datetime* in the MATLAB) of the first data point; and (5) *file*, which specifies the full path to the file.

To facilitate the integration of this toolbox with results from other automatic detector toolboxes, HFOApp can read output events that are organized as a MATLAB structure array that contains the fields *label* and *info*. The *label* field indicates the channel name, and *info* is an *N* × three cell array, where *N* is the number of events for the channel. The first column of the cell array *info* indicates the location of events, and the second column indicates the type of events. The event type must be one or more of the following: *γ*, *ripple*, *fastripple*, *ultrafast*, and *spike*. The last column of *info* is a data structure containing the following fields: *AvgFreq* (average frequency), *NoCycles* (number of cycles of the oscillation), *PeakZscore* (maximal *z* score of the event), and *BPFreq* (bandpass frequency band). Once the events are organized in this format, they can be loaded into HFOApp for reviewing or further editing. This feature makes HFOApp a useful visualization and verification tool for the review of results of other automatic detector tools.

The output of HFOApp is simply two text files. Within one file, detailed information about all events from all channels are organized as a formatted table (Fig. 2*C*). The user can also organize their own events in this format to be loaded into HFOApp for visual validation. The second file is a summary file, which indicates the event information of each channel (e.g., number of occurrences of each event type, total duration), which is useful for a quick glimpse at the results (Fig. 2*D*).

### Graphical user interface layout

**Figure 3.**
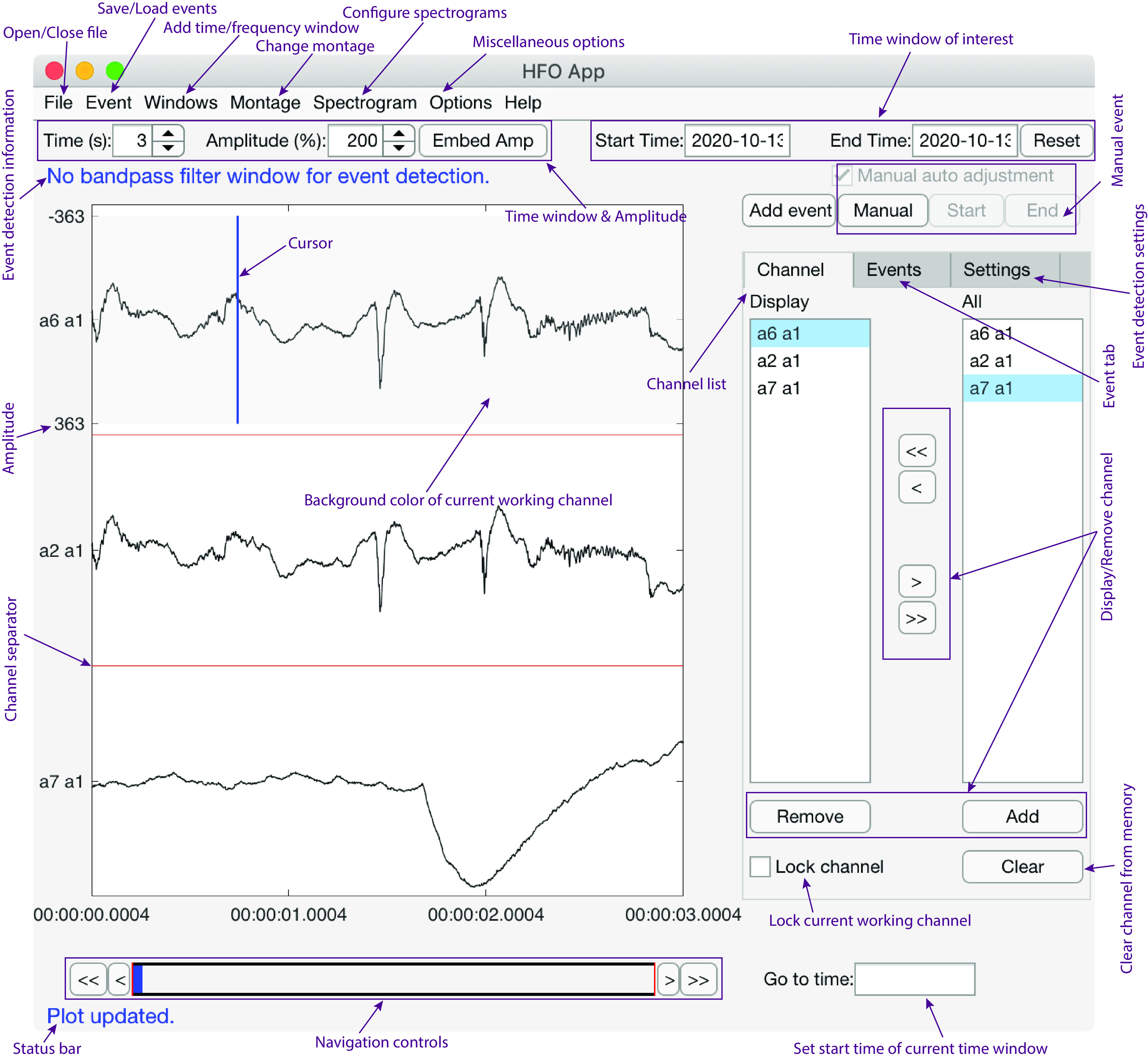
Features of the time series graphical user interface of HFOApp. The main window and bandpass-filter window share the same graphical user interface.

**Figure 4.**
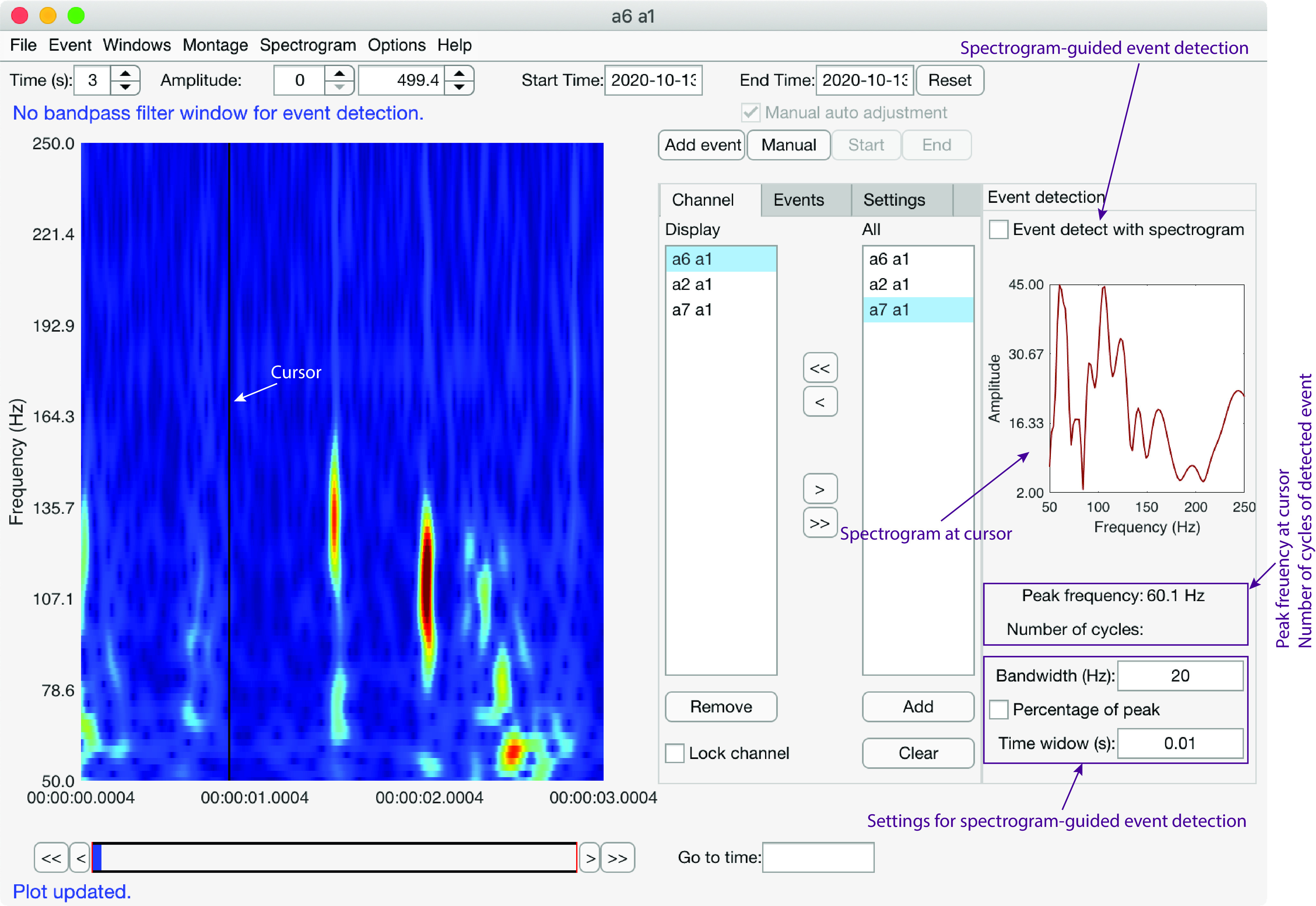
Features of the spectrogram graphical user interface of HFOApp. The layout of the spectrogram window is similar to the main window, with an extra panel added for spectrogram event detection.

HFOApp displays time (Fig. 3) and spectrogram (Fig. 4) domain windows. The bandpass-filter time window shares an identical graphical user interface with the main window. The user can filter the raw data into multiple different frequency bands, and each resulting time series can be displayed either in a separate window or embedded in the main window.

To specify a channel to mark, the user simply clicks on the time series of a channel. The background of that channel will be highlighted, and the user can then mark events on that channel. All relevant information pertaining to the location of the click within that time series is immediately displayed in the status bar (Fig. 3, status bar, event detection information). No menu-based setting or switching is required. We believe this feature significantly reduces the time needed to mark HFOs on a dataset with multiple channels. HFOApp was designed to minimize the number of pop-up windows to speed navigation and improve the user experience.

The spectrogram window looks similar to the main window (Fig. 4). By default, only one spectrogram window is open at a time to conserve speed. However, it is possible to open multiple spectrogram windows, which is advisable only if the file size of the dataset is small. When the “Event detection with spectrogram” box (Fig. 4) is checked, the user can perform spectrogram-guided automated event detection by clicking on any hotspot in the spectrogram, as described above.

### Implementation of the Hilbert detector

**Figure 5.**
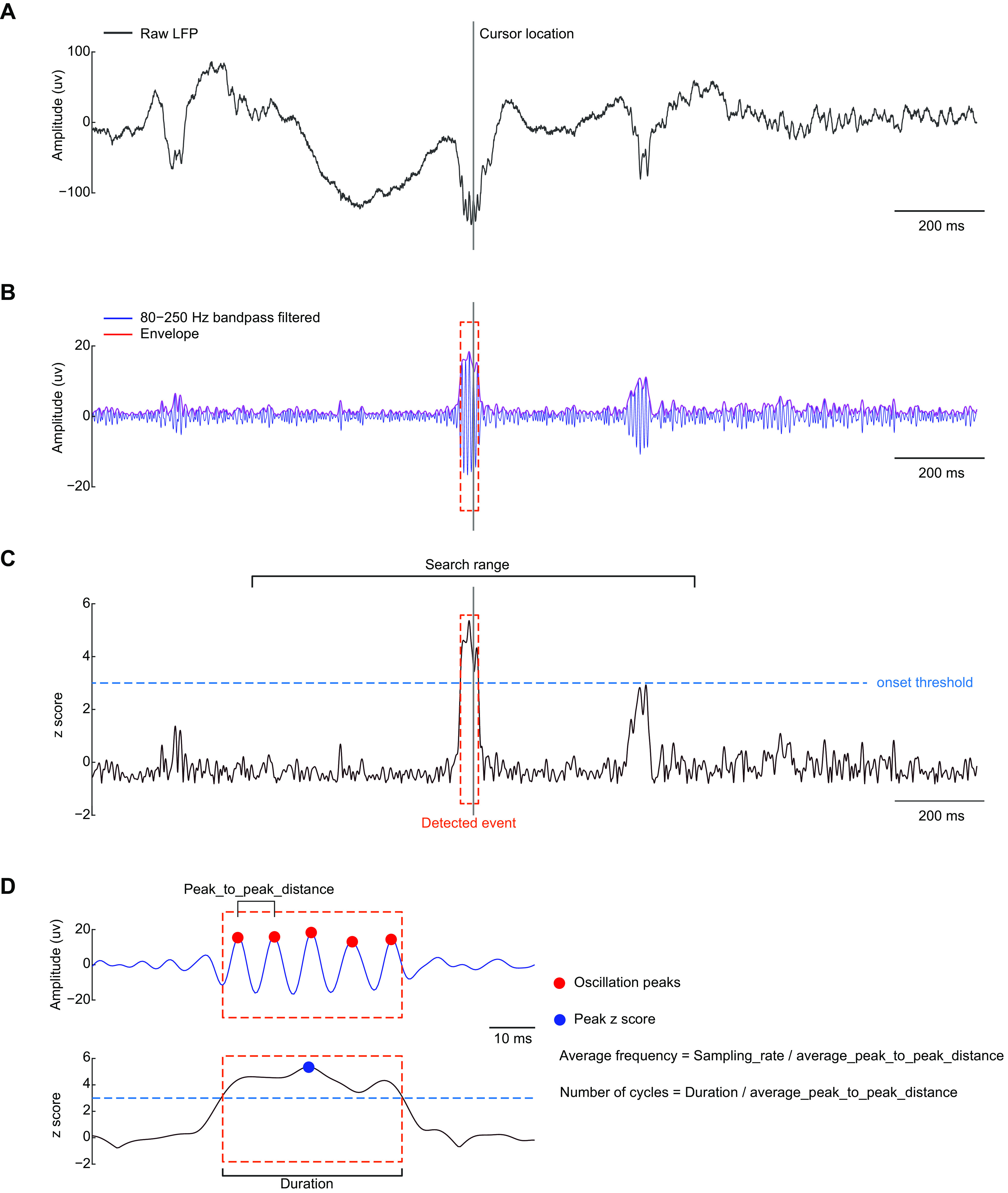
Schematic plot of Hilbert detector implementation. ***A***, An example of raw iEEG data containing HFOs. The vertical gray line indicates cursor location. ***B***, Bandpass filtered data (blue) and its envelope (red), obtained using the Hilbert method. ***C***, HFO detection. The envelope time series (***B***) is first *z* score normalized. Clusters surviving an initial threshold (onset threshold) within a distance (search range) of the cursor location are identified. The cluster will be considered an HFO event if the following two criteria are met: (1) the maximal *z* score is greater than a user-specified threshold (in this example 5); and (2) the number of cycles meets the user-specified value (in this example 3). ***D***, Characteristics of an event. For a detected event (red dashed rectangle), the following characteristics are retrieved: peak *z* score (blue dot), duration, average frequency, and number of cycles. To estimate the average frequency, oscillatory peaks for the event are identified (red dots) and all peak-to-peak distances are calculated, then the sampling rate is divided by the average peak-to-peak distance. The number of cycles is estimated by dividing the duration of the event by the average peak-to-peak distance. The red dashed boxes in ***B–D*** indicate an HFO event.

The default automated detector in HFOApp is custom scripted using a Hilbert detector (Crépon et al., 2010; Höller et al., 2018). This detector is used by default for fully automated detection and is used in the interactive automation-assist features. In detail, the raw time series is bandpass filtered at a specified frequency band. A notch filter can also be applied to remove 50/60 Hz power line noise. Then, the envelope of the bandpass-filtered time series is obtained using the Hilbert transform (MATLAB *hilbert.m*; Fig. 5*A*,*B*). Finally, the envelope is *z* score normalized to a baseline, which can be the entire time series or a time window of specified length (Fig. 5*C*). Normalization is performed by subtracting the mean of the baseline and further dividing by the SD of the baseline. By default, the toolbox uses the filter functions implemented in the *fieldtrip* toolbox to perform bandpass filtering. For both bandpass and notch filtering, the user can choose to use either a fourth order of Butterworth (IIR) filter or a window-based finite impulse response (FIR) filter, which uses the MATLAB *fir1* function. The default FIR filter order is three times the sample rate, which is further divided by the low cutoff frequency of the bandpass filtering. However, the user can also implement their own filtering procedure in *HFOFiltData.m*. To characterize an event, the toolbox calculates the following features: (1) average frequency, (2) number of cycles, and (3) maximal *z* score (Fig. 5*D*). To calculate the average frequency, the peaks of the bandpass-filtered time series are located within the event time window (MATLAB *findpeak.m*). Then the average frequency is calculated by dividing the sampling rate by the average peak-to-peak distance. The number of cycles is obtained by dividing the duration of the event by the average peak-to-peak distance.

Note, HFOApp has two different Hilbert detectors built in: one introduced above, and the other from RIPPLELAB (Navarrete et al., 2016). The default Hilbert detector in HFOApp is different from that in RIPPLELAB. In RIPPLELAB, the Hilbert detector uses three parameters, including epoch length, SD threshold, and minimal duration, to detect HFOs. In HFOApp, we use two SD thresholds—onset and peak thresholds—instead of one single threshold. Furthermore, in HFOApp the duration of an HFO event is determined by the number of cycles instead of duration in milliseconds.

### Implementation of the spectrogram

**Figure 6.**
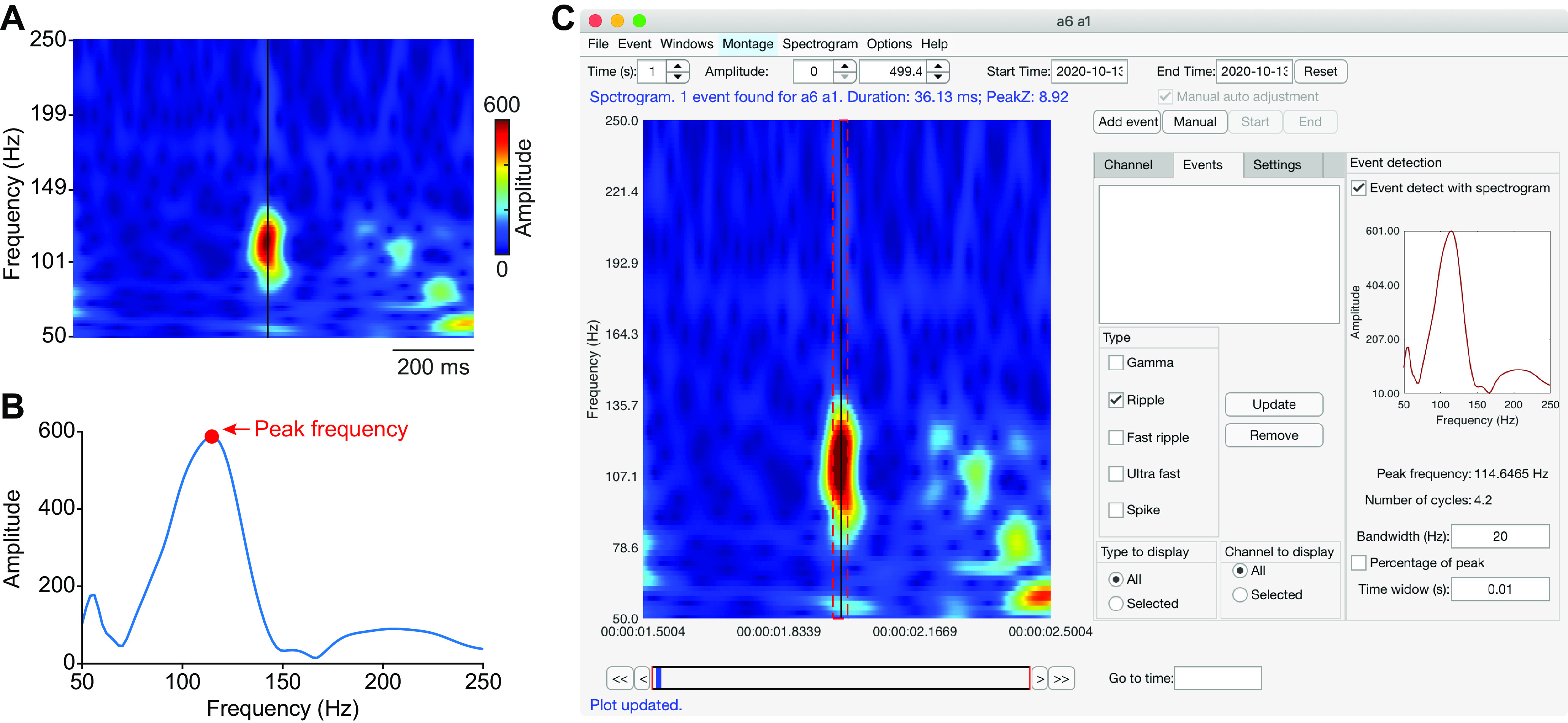
HFO detection using spectrogram. ***A***, In the spectrogram detection mode, the user clicks on the hotspot of the spectrogram. The black vertical line indicates the location of the click. ***B***, The toolbox computes the peak frequency (red dot) of the spectrogram at the cursor location (***A***). The HFO is then identified using the Hilbert detector with a narrow bandwidth, which can be specified by the user, centered at the peak frequency. ***C***, A screenshot of the spectrogram window of the toolbox. The black vertical line indicates the cursor location, and the red dashed rectangle indicates the detected HFO.

To increase the sensitivity of the Hilbert detector, we include a feature to allow bandpass filtering of the raw time series at a narrow frequency band. To do this, we first calculate the time–frequency spectrogram (Fig. 6*A*) using a Wavelet transform calculated using a continuous Gabor wavelet (Navarrete et al., 2016). The wavelet is defined as follows:

$$C(s\text{,}t)=\frac{1}{\sqrt{s}}\int_{-\infty }^{\infty }x(t)*\text{ψ}(\frac{t\text{-τ}}{s})\text{dt},\cdot \cdot \cdot \cdot \text{ψ}(t)=\frac{1}{{\left(\sigma^{2}\Pi \right)}^{\frac{1}{4}}}\text{ exp}(\frac{-{\text{t}}^{2}}{2\sigma^{2}})e^{{\text{iη}}_{\text{s}}t},$$

where the *t*, *s*, and τ represent time, scale, and translation, respectively. $\eta_{s}$ is the angular frequency at *s*. σ (default value of 6/ $\eta_{s}$), indicates the SD of the Gaussian window in time (Navarrete et al., 2016).

The user can modify *HFOSpectrogram.m* to implement their preferred method of spectrogram computation. To begin the spectrogram-guided HFO detection feature, the user clicks on the hotspot of interest in the spectrogram. The spectrogram at the cursor location will be extracted and averaged over a small time window centered at the cursor, and the peak frequency will be computed (Fig. 6*B*). The length of the small time window can be changed by the user in the spectrogram user interface [Fig. 6*C*, Time window (s)]. Then, the raw time series will be bandpass filtered over a narrow frequency band centered at the peak frequency, and the HFO event will be detected and marked using the method described above (Fig. 6*C*). The bandwidth can be set as a fixed value [e.g., 20 Hz; Fig. 6*C*, Bandwidth (Hz)], but the user can also set the cutoff frequency based on the peak spectrogram. If the “Percentage of peak” box (Fig. 6*C*) is selected, the “Bandwidth (Hz)” will be interpreted as a percentage that ranges from 0% to 100%, and the frequencies at which the spectrogram (Fig. 6*B*) drops to the specified percentage of the maximum will be used as cutoff frequencies for the bandpass filtering.

### Simulated datasets for validation

The simulated datasets used in the validation experiments were generated using Python toolboxes MNE (RRID:SCR_005972) and MNE-HFO [Gramfort et al., 2013; see also “MNE-HFO: an open-source Python implementation of HFO detection algorithms” (https://zenodo.org/record/4485036)]. For each simulated dataset, sinusoidal data of different frequencies (2.5, 6.0, 10.0, 16.0, 32.5, 67.5, 165.0, 250.0, 425.0, 500.0, 800.0, and 1500.0 Hz) were added together, and a few HFOs were inserted into the simulated time series. The sampling rate was set to 2000 Hz. The frequencies of the simulated HFOs were set to 100, 140, 180, and 220 Hz, each consisting of 20 events. The number of cycles for each HFO was a random integer between 3 and 10. Then, we added random noise to the simulated data such that the signal-to-noise ratio ranged from 1 to 10, in steps of 1. In total, we generated 110 datasets with varying levels of noise. Finally, the HFOs of the simulated datasets were detected using both HFOApp and RIPPLELAB, using the default Hilbert detector of each program. For HFOApp, the onset threshold, inclusion threshold, and number of cycles were set to 1, 5, and 2.4, respectively. For RIPPLELAB, the threshold and minimal duration were set to 3.5 and 10 ms, respectively. These parameters were chosen to detect HFOs with a minimum of four cycles, given the characteristics of the simulated dataset. The epoch length was set to 600 s for both HFOApp and RIPPLELAB Hilbert detectors.

### Real datasets for validation experiments

The real datasets were recorded using a clinical acquisition system (Blackrock) with a 4 × 5 grid (Integra). This dataset was part of a large study that was approved by the Northwestern University Institutional Review Board. To manually mark HFO events, the data were first bipolar rereferenced and organized into the data format required by either HFOApp or RIPPLELAB. Two channels were analyzed per dataset. The raters were instructed to manually mark ripples (frequency band, 80–250 Hz) using a bandpass-filtered time series between 80 and 250 Hz. Raters used the default HFOApp manual marking setup, which includes interactive automation-assist features (Fig. 1*A–C*), and in RIPPLELAB they followed the instructions for manual marking in the manual of the toolbox.

## Results

### Validation of HFOApp

We conducted a series of experiments to validate HFOApp. In a first validation experiment, we tested how accurately the HFOApp automated Hilbert detector could detect HFOs in a simulated dataset that provided known, ground-truth HFO events. In a second validation experiment, we compared the performance of HFOApp with that of a previously existing HFO marking software, RIPPLELAB (Navarrete et al., 2016), on the same simulated dataset. In a third validation experiment, we used real data to test whether HFOApp improves manual marking speed and inter-rater variability over previously existing methods.

**Figure 7.**
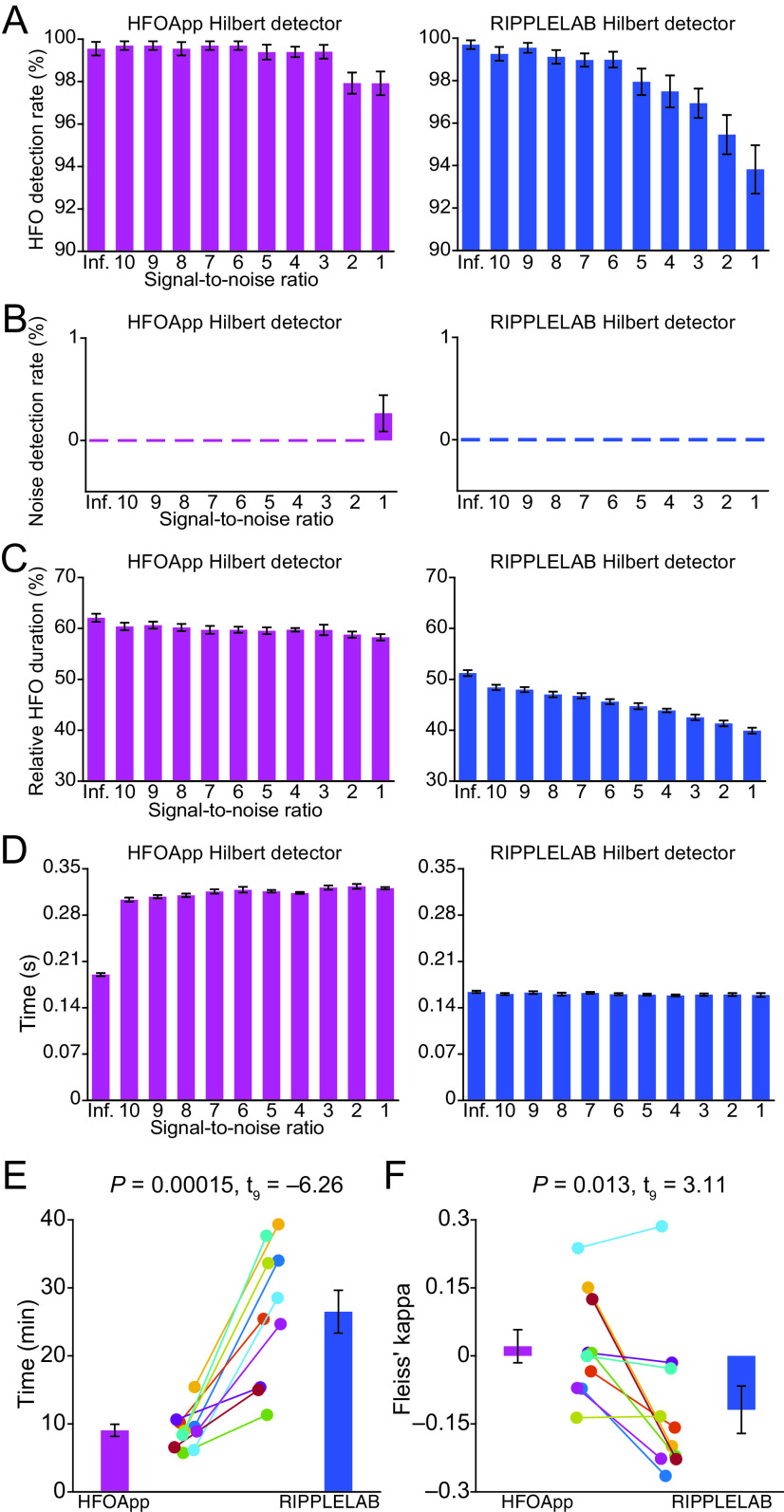
Validation of HFOApp. ***A***, Proportion of simulated HFOs that were detected by HFOApp and RIPPLELAB default automatic Hilbert detectors. ***B***, Proportion of detected HFOs that were not real simulated HFOs. ***C***, Average duration of detected HFO relative to that of the simulated HFO. ***D***, Computation time. ***E***, Manual marking time for 10 real datasets. ***F***, Inter-rater reliability (Fleiss’ κ) across four raters for the real datasets. The error bars in ***A–D*** indicate the SE of the average across 10 simulated datasets. The error bars in ***E*** and ***F*** indicate the SE of the average across 10 patients. The comparison of time and Fleiss’ κ values between HFOApp and RIPPLELAB was performed using a two-tailed paired *t* test.

In the first validation experiment, we tested how accurately the HFOApp automated Hilbert detector could detect HFOs in a simulated dataset which provided ground-truth HFO events. This allowed us to confirm that the HFOApp automated detector performs as intended. This validation was conducted on 110 simulated datasets. Each dataset was 10 min long and contained 80 HFO events. Datasets were generated to contain HFO events of between 3 and 10 cycles, and frequencies ranging from 100 to 220 Hz. To assess the ability of HFOApp to detect HFOs within a noisy environment, the simulated datasets had 10 different levels of background noise, ranging from a signal-to-noise ratio of 1 to a signal-to-noise ratio of 10; 10 files were created for each noise level. HFO detection was set to include events with four or more cycles. We found that HFOApp detected events with accuracy ranging from 99.7% at the lowest noise level, to 97.9% at the highest noise level (Fig. 7*A*, left).

**Figure 8.**
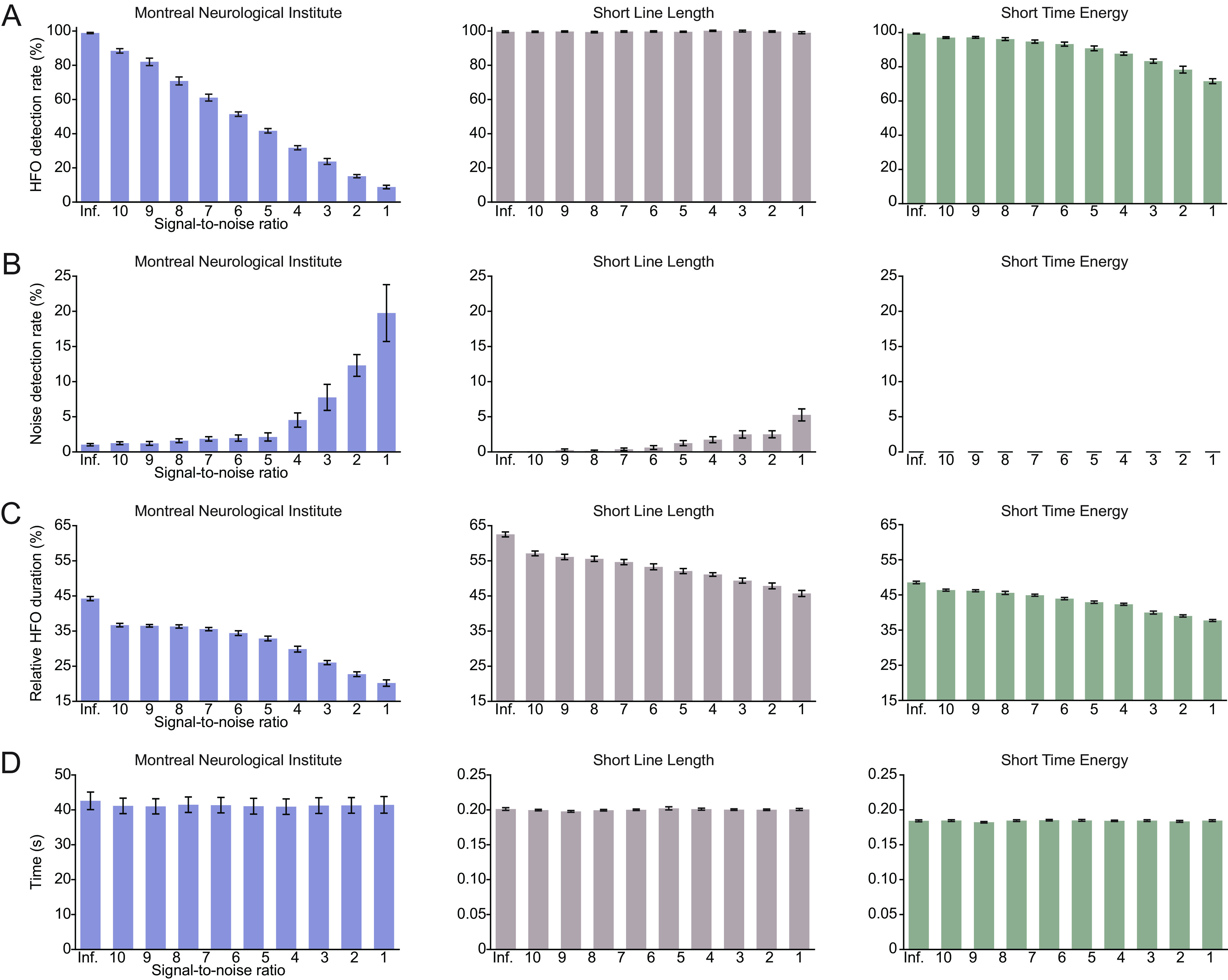
Automatic HFO detection results of the simulated dataset. ***A***, Proportion of simulated HFOs that were detected by the Montreal Neurologic Institute detector (left column), the Short Line Length detector (middle column**)**, and the Short Time Energy detector (right column). ***B***, Proportion of detected HFOs that were not real simulated HFOs. ***C***, Average duration of the detected HFO relative to that of the simulated HFO. ***D***, Computation time. The error bars in ***A–D*** indicate the SE of the average across 10 simulated datasets.

In the second validation experiment, we compared the HFOApp results to those of existing software by using the RIPPLELAB automated Hilbert detector to detect HFOs in the same simulated dataset. This allowed us to confirm that HFOApp is able to detect HFOs with an accuracy similar to those of other detectors that are currently available. We analyzed the results across the two applications at varying noise levels by comparing detection of real events, detection of false events, and accuracy of detected event duration. The two applications detected real events at a similar rate across noise levels, though HFOApp slightly outperformed RIPPLELAB (Fig. 7*A*,*C*, left and right columns). RIPPLELAB accuracy ranged from 99.7% at the lowest noise level, to 93.8% at the highest noise level (Fig. 7*A*, right). The HFOApp detection rate of false events over the simulated datasets, defined as the number of nonsimulated HFOs divided by the number of total detected events, was similar to that of RIPPLELAB (Fig. 7*B*). Detected HFO duration relative to the real duration was more consistent across different levels of signal-to-noise ratio in HFOApp than in RIPPLELAB (Fig. 7*C*). As expected, the detection time is slightly longer in HFOApp than in RIPPLELAB, as more computations are involved in HFOApp (Fig. 7*D*). Overall, these analyses confirmed that the HFOApp automated detector performs similar to or better than RIPPLELAB in accurately and consistently detecting HFO events across different levels of noise. Figure 8 indicates the performance of other automatic detectors in HFOApp. including the Montreal Neurologic Institute detector (Fig. 8*A–D*, left column), the Short Line Length detector (Fig. 8*A–D*, middle column**)**, and the Short Time Energy detector (Fig. 8*A–D*, right column). The parameters for each detector, which can be found in the toolbox, were optimized for maximal detection on the simulated data without noise.

In the third validation experiment, we tested improvements in the manual marking speed and inter-rater variability of HFOApp. To do this, we asked four expert raters to manually mark HFO events in 10 two-channel, 3 min, real datasets from surgical patients. Each rater performed manual marking using both HFOApp and RIPPLELAB. Raters used HFOApp default manual marking settings, which include the interactive automation-assist features. This resulted in 40 manually marked datasets for both HFOApp and RIPPLELAB. We then compared marking time and inter-rater variability across the two different applications. We found that HFOApp markings were statistically significantly faster, and that HFOApp produced significantly reduced inter-rater variability (Fig. 7*E*,*F*). To calculate the manual marking time, we averaged the time across raters for each dataset and each toolbox. The results indicated that the average time for marking two channels was 9.04 ± 0.89 min for HFOApp and 26.49 ± 3.14 min for RIPPLELAB. The data are presented as the mean ± SE unless stated otherwise. The time difference was statistically significant, as revealed by a two-tailed paired *t* test (*p *=* *0.00,015, *t*_(9)_ = –6.26; Fig. 7*E*). To evaluate inter-rater variability, we calculated the Fleiss κ value using a MATLAB toolbox developed by Giuseppe Cardillo (https://github.com/dnafinder/Fleiss). Our results indicated improved Fleiss’ κ values for HFOApp (0.021 ± 0.037), compared with RIPPLELAB (–0.119 ± 0.052), suggesting that HFOApp has statistically higher inter-rater reliability (two-tailed paired *t* test; *p *= 0.013, t_9_ = 3.11; Fig. 7*F*). We note that Fleiss’ κ is not ideal for indexing inter-rater agreement measures for HFO detection, but was used here as a rough measure, and for lack of a better alternative (Zelmann et al., 2009; Menendez De La Prida et al., 2015; Navarrete et al., 2016). Fleiss’ κ is typically used in the psychological and psychiatric fields, and is designed to assess the reliability of agreement between raters who assign ratings within a set number of exclusive categories. HFO detection in electrophysiological data are a poor fit for Fleiss’ κ, as it does not have a set number of exclusive categories for rating, lacks a reference—or ground-truth—interpretation, and is a difficult EEG interpretation category, with typically low signal-to-noise ratios. Rater agreement in the interpretation of such data, when measured with Fleiss’ κ, has been found to be typically low (Gerber et al., 2008; Grant et al., 2014). As expected, both programs scored on the low end of the Fleiss’ κ index, but HFOApp scored significantly higher than RIPPLELAB. Together, these findings suggest that HFOApp is significantly faster, with higher inter-rater reliability, two particularly significant advantages for clinicians and researchers who are manually reviewing and marking iEEG data.

## Discussion

In this article, we introduced HFOApp, a MATLAB graphical user interface for manual marking of HFOs in intracranial electroencephalographic recordings. The advantages of this toolbox, compared with existing software, include ease of use, comprehensibility of design, flexibility of implementation, optional automation-assist for on-the-fly use at multiple levels of manual marking, and an architecture built on an active, supported platform, making HFOApp a versatile and powerful HFO marking tool, with significant improvements over existing applications. We believe that these features will make HFOApp a useful tool for clinicians and researchers who study HFOs.

## Synthesis

Reviewing Editor: William Stacey, University of Michigan Decisions are customarily a result of the Reviewing Editor and the peer reviewers coming together and discussing their recommendations until a consensus is reached. When revisions are invited, a fact-based synthesis statement explaining their decision and outlining what is needed to prepare a revision will be listed below. The following reviewer(s) agreed to reveal their identity: Richard Staba, Catalina Alvarado-Rojas.

We feel this is a clear improvement in the paper, but there are still several concerns that should be addressed. We have listed them below. Most are suggestions to improve presentation, but the most important items are #1 (is there a way to make this more universal across matlab versions?), #5/6 (need to explain more about the interrater variability), and #7 (need to show performance for more methods).

1. neither reviewer could open the file in their versions of Matlab, and thus were unable to test the files. While this makes it difficult to review, it creates even more of a concern for readers of the article. We strongly suggest making it less dependent upon version-specific functions. The fact that two independent experts, who might be potential users of this tool, are unable to use it simply for having the wrong version of software is a major concern.

2. in the visual abstract, we suggest including showing multiple channels simultaneously,to highlight that feature.

3. There is a comparison of the marking time for visual marking. But it would be interesting to compare the computation time for automatic detection between RippleLab and HFOApp.

4. Automatically saving results in HFOApp is useful. The manuscript should indicate if the HFOApp has an undo function/shortcut key or whether an event added in error has to be deleted.

5. Regarding the inter -rater reliability, scoring of HFOs was more reliable using HFOApp than RippleLab, yet with either program reliability was poor. Please clarify if this correct and if it is, it would be helpful to explain the poor performance in detecting HFOs.

6. What is the explanation for the Fleiss’ kappa so close to 0 for both detectors?

7. There is no comparison among the implemented methods besides the Hilbert Detector. It would be helpful to evaluate and compare the correct detection rates of the other 3 automatic detectors.

8. During the HFO detection process, users may prefer to have the signal, the filtered signal and the time-frequency plot in the same figure, rather than three separate windows.

9. Introduction line 72: artifactual spike as well as epileptiform EEG spikes

10. Abstract line 14: The use of “epileptogenic tissue", as written in the 1. Introduction line 58, seems more appropriate in the context of epilepsy surgery rather then the currently worded “epileptogenesis"

## Author Response

Synthesis of Reviews:

Computational Neuroscience Model Code Accessibility Comments for Author (Required):

A link to access HFOApp should be in the text more prominently

Thank you. We have added a more prominent link to the clean (not change-tracked) version of the manuscript. This link does not appear in the reviewers’ version in order to comply with double-blind review requirements.

Significance Statement Comments for Author (Required):

N/A

Comments on the Visual Abstract for Author (Required):

we suggest including showing multiple channels simultaneously,to highlight that feature.

Thank you, we have made this change. Please see the response to #2, below.

Synthesis Statement for Author (Required):

We feel this is a clear improvement in the paper, but there are still several concerns that should be addressed. We have listed them below. Most are suggestions, but the most important items are #1 (is there a way to make this more universal across matlab versions?), #5/6 (need to explain more about the interrater variability), and #7 (need to show performance for more methods).

1. neither reviewer could open the file in their versions of Matlab, and thus were unable to test the files. While this makes it difficult to review, it creates even more of a concern for readers of the article. We strongly suggest making it less dependent upon version-specific functions. The fact that two independent experts, who might be potential users of this tool, are unable to use it simply for having the wrong version of software is a major concern.

We appreciate the reviewers’ concern about HFOApp’s compatibility with earlier Matlab versions. HFOApp’s GUI was created using the uifigure function, which is used by App Designer, which was introduced by Matlab in 2016 and is their recommended environment for building apps. The functionalities of uifgure have been under development and vastly expanded since its introduction, and are supported in concurrent versions of Matlab. As they develop App Design, Matlab limits backwards compatibility, which limits developers’ ability to make apps compatible with earlier versions of Matlab. We have tried very hard to find a way to work around this limitation while still maintaining HFOApp’s fundamental features. However, some essential functionalities are not supported by earlier version of the platform For example, WindowButtonDownFcn, which defines the mouse click response required by HFOApp, is not available in earlier releases (R2016a,b, R2017a,b, R2018a). In R2018b, the function that is needed to get the position of the mouse click on the time series or navigation bar, i.e. the CurrentPoint property of the axes, is not available. These limitations have unfortunately made HFOApp incompatible with Matlab versions earlier than R2019a. We have been able to run HFOApp on Matlab versions of R2019a,b, R2020a,b and R2021 without issues.

While we acknowledge this limit, it is important to note that the newer functions that Matlab has developed in App Designer have allowed us to design HFOApp to include features that allow for usability and functional improvements based on updates and advances that Matlab made in their interactive functions. In order to create a version of HFOApp that is compatible with earlier Matlab versions, we would need to design an almost entirely new software using the prior environment, Guide, which is against Matlab’s recommendations for new app development, would not be able to include the usability features and improvements that we integrated into HFOApp, and would thus not provide a consistent user experience with the current version.

We respectfully ask reviewers to consider the fact that HFOApp is making use of some of the newest features available in Matlab, and is therefore at the cutting edge of what the program is capable of doing. Unfortunately, the functionalities of uifigure are not supported by old Matlab versions, and the older functions would not achieve a similar level of functionality. Most universities provide Matlab licenses with access to current versions of Matlab at no additional cost to the end user. We therefore expect that most researchers and clinicians will be able to obtain access to current versions of Matlab, and thus HFOApp’s being up-to-date with current versions will not be a major limitation for most labs. In addition, this problem will fade as older versions of Matlab decline in use. We respectfully point out that keeping software current is not an unreasonable burden to researchers and clinicians involved in sensitive EEG analysis, and we expect that most users interested in HFOApp will have already done so.

We have added language specifying Matlab version requirements to the Methods. In addition, Matlab version requirements are specified in the System Requirements section of HFOApp’s manual, which is provided in the toolbox as a PDF. We have updated this language, and it now reads: “Windows/Mac/Linux MATLAB versions R2019a,b, R2020a,b, R2020b, or R2021a.” We will update HFOApp for new releases of Matlab, and will note these updates on our release page.

Below is a summary of the compatibility with each version of Matlab (Required toolbox: Signal processing and Statistics and Machine Learning) that we tried:

R2016a,b

>> HFOApp

No public property AutoResizeChildren exists for class matlab.ui.Figure.

Error in HFOApp/createComponents (line 7926)

app.HFOAppUIFigure.AutoResizeChildren = ‘off’;

Error in HFOApp (line 8595)

createComponents(app)

R2017a,b

>> HFOApp

Functionality not supported with figures created with the uifigure function. For more information,

see Graphics Support in App Designer.

Error in HFOApp/createComponents (line 7854)

app.HFOAppUIFigure.WindowButtonDownFcn = createCallbackFcn(app,

@HFOAppUIFigureWindowButtonDown, true);

Error in HFOApp (line 8517)

createComponents(app)

R2018a

>> HFOApp

Functionality not supported with figures created with the uifigure function. For more

information, see Graphics Support in App Designer.

Error in HFOApp/createComponents (line 7854)

app.HFOAppUIFigure.WindowButtonDownFcn = createCallbackFcn(app,

@HFOAppUIFigureWindowButtonDown, true);

Error in HFOApp (line 8517)

createComponents(app)

R2018b

>> HFOApp

No appropriate method, property, or field ‘Toolbar’ for class ‘matlab.ui.control.UIAxes’.

Error in HFOApp/createComponents (line 8498)

app.UIAxes.Toolbar.Visible = ‘off’;

Error in HFOApp (line 8517)

createComponents(app)

The above ‘Toolbar’ error can be solved by commenting line 8498. However, the user will then encounter the error below. This error is due to the release version, it doesn’t seem to be fixable.

>> HFOApp

No appropriate method, property, or field ‘CurrentPoint’ for class

‘matlab.ui.control.UIAxes’.

Error in HFOApp/HFOAppUIFigureWindowButtonDown (line 7668)

app.UIAxes.CurrentPoint( 1, 1:2),...

Error while evaluating Figure WindowButtonDownFcn.

2. in the visual abstract, we suggest including showing multiple channels simultaneously,to highlight that feature.

Thank you for the great suggestion. We have revised the visual abstract to show multiple channels.

3. There is a comparison of the marking time for visual marking. But it would be interesting to compare the computation time for automatic detection between RippleLab and HFOApp.

Thank you for this good suggestion. We have added the results of computation time to the manuscript on page 35. The computation time is now shown in a new version of Fig. 7D,

4. Automatically saving results in HFOApp is useful. The manuscript should indicate if the HFOApp has an undo function/shortcut key or whether an event added in error has to be deleted.

Thank you for this suggestion. If an event was added to the Events List by mistake, the user can select the event and press the Remove button below the Events List. The results will be automatically updated whenever an event is added or removed from the Events list if automatic saving was enabled. We have highlighted this feature in manuscript on page 15.

5. Regarding the inter -rater reliability, scoring of HFOs was more reliable using HFOApp than RippleLab, yet with either program reliability was poor. Please clarify if this correct and if it is, it would be helpful to explain the poor performance in detecting HFOs.

6. What is the explanation for the Fleiss’ kappa so close to 0 for both detectors?

These are good questions.

To clarify, the two programs scored in two different Fleiss’ kappa categories, as defined Landis and Koch (1977), with HFOApp scoring higher: RippleLab showed a kappa index of “poor", while HFOApp showed a kappa score of “slight”. This analysis deserves some further explanation, and we are grateful for the reviewers’ bringing our attention to it.

There are two main points to be made in response to these questions. First, there is no good, established way to measure inter-rater reliability for HFO marking, and second, it is well-known that HFO marking varies widely across raters. More broadly speaking, the difficulty of assessing inter-rater reliability in the interpretation of electrophysiological data is an often-discussed problem, which is due to the lack of a true or ground-truth interpretation (Grant et al 2014, Gerber et al 2008). EEG interpretation rater agreement, when measured with Fleiss’ kappa, has been found to be typically low when the interpretive category is particularly difficult. This is the case with interpretation of HFO events. In this context, our finding of a kappa score indicating slight inter-rater reliability with HFOApp is a note-worthy improvement over a kappa score indicating poor inter-rater reliability with RippleLab.

See references below:

Gerber PA, Chapman KE, Chung SS, Drees C, Maganti RK, Ng Y, Treiman DM, Little AS, Kerrigan JF (2008) Interobserver agreement in the interpretation of EEG patterns in critically Ill adults. J Clin Neurophysiol 25:241-249.

Grant AC, Abdel-Baki SG, Weedon J, Arnedo V, Chari G, Koziorynska E, Lushbough C, Maus D, McSween T, Mortati KA, Reznikov A, Omurtag A (2014) EEG interpretation reliability and interpreter confidence: A large single-center study. Epilepsy Behav 32:102-107.

Landis J, Koch G (1977) The measurement of observer agreement for categorical data. Biometrics 33:159-174.

Menendez De La Prida L, Staba RJ, Dian JA (2015) Conundrums of high-frequency oscillations (80-800 Hz) in the epileptic brain. J Clin Neurophysiol.

Navarrete M, Alvarado-Rojas C, Le Van Quyen M, Valderrama M (2016) RIPPLELAB: A comprehensive application for the detection, analysis and classification of high frequency oscillations in electroencephalographic signals. PLoS One 11.

Zelmann R, Zijlmans M, Jacobs J, Châtillon CE, Gotman J (2009) Improving the identification of High Frequency Oscillations. Clin Neurophysiol 120:1457-1464.

Since there is not a well-established method for measuring inter-rate reliability for HFO markers, we reasoned that the best way to respond to the reviewers’ previous request was to focus on showing that HFOApp has improved inter-rater reliability over an existing app, but not to be concerned with the overall levels of inter-rater reliability, which we expected to be low, given previous studies and the nature of the task of identifying HFOs, which are low signal-to-noise ratio events in EEG data. The measure we used, Fleiss’ Kappa, is typically used in the psychological and psychiatric fields, and is designed to assess reliability of agreement between raters who assign ratings within a set number of exclusive categories. Therefore it is not ideal for indexing agreement in interpretation of EEG data. However, for lack of a better alternative, it has been used in EEG literature, though with caveats. In our case, though the values on the scale do not carry the intended meaning when used for HFO marking, and lack the sensitivity to fully index agreement between our raters, we nonetheless used this scale as a simple way to compare the values across HFOApp and RippleLab. We were not surprised that the inter-rater reliabilities were overall low for both toolboxes using this index, but note that HFOApp performed a category above RippleLab. But the important conclusion to be taken from this analysis is that HFOApp shows significant improvement over RippleLab. We have added a discussion of this point to the manuscript on page 36.

7. There is no comparison among the implemented methods besides the Hilbert Detector. It would be helpful to evaluate and compare the correct detection rates of the other 3 automatic detectors.

Thank you for the suggestion. We agree that it would be informative to some readers to show the performance of other automatic detectors on the same simulated datasets. We have added the HFOs detection results of other detectors to new Figure 8.:

8. During the HFO detection process, users may prefer to have the signal, the filtered signal and the time-frequency plot in the same figure, rather than three separate windows.

Thank you for pointing this out. In HFOApp, the user can display the raw signal and filtered signal as different rows in the same window, and can add as many filtered windows as they want. When creating a filtered time-series, the user simply unchecks the “Open in new Window” box in order to display the filtered data right below the raw data in the same window. We have mentioned this feature in the section “Summary of improvements over existing open-source HFO applications” on page 7 and also in “Using HFOApp” on page 14. As far as the time-frequency plot, we chose to place it in a new window in consideration of plotting speed. But the user can easily arrange the time-frequency window just below the main window if they prefer to do so, because all figures, including all cursors, are synchronized.

9. Introduction line 72: artifactual spike as well as epileptiform EEG spikes

This is a good point. We have revised “artifactual spike” to “artifactual spike as well as epileptiform EEG spikes”.

10. Abstract line 14: The use of “epileptogenic tissue", as written in the 1. Introduction line 58, seems more appropriate in the context of epilepsy surgery rather then the currently worded “epileptogenesis"

Thank you for the suggestion. We have changed “epileptogenesis” to “epileptogenic tissue”.
