# Supplementary material for: HFOApp: A MATLAB Graphical User Interface for High-Frequency Oscillation Marking
Source: eNeuro. 2021 Oct 8;8(5):ENEURO.0509-20.2021. doi: 10.1523/ENEURO.0509-20.2021 (PMC8503963; doi:10.1523/ENEURO.0509-20.2021)
Supplement: Extended Data 1 — HFO App Package V1. Download Extended Data 1, ZIP file. [file enu-eN-MNT-0509-20-s02.zip › HFOApp_Package_V1/HFOApp_Manual.pdf]

HFOApp—High Frequency Oscillations Application

High Frequency Oscillations Application (HFOApp) is a MATLAB-based graphical user interface designed to optimize visual marking of high frequency oscillations in intracranial electroencephalography recordings.

System requirement:

- Windows/Mac/Linux MATLAB versions R2019a,b, R2020a,b, or R2021a.
- MATLAB Signal Processing and Statistics and Machine Learning Toolbox.
- Screen resolution better than 1200 x 800. Minimum requirement: 900 x 600.

Development environment: App Designer, MATLAB R2020b, MacOS Mojave (Version 10.14.6). HFOApp may not work on a MATLAB version earlier than 2019a.

Note. HFOApp uses *fieldtrip* toolbox **(Oostenveld et al., 2011)** to perform bandpass filtering and functions derived from RIPPLELAB **(Navarrete et al., 2016)** to perform time-frequency decomposition. However, installation these toolboxes is not required because all relevant functions are included in the distribution package of HFOApp.

Version 1.0. July 12, 2021.

Table of Contents

SUMMARY *1*

KEYBOARD SHORTCUTS *2*

DATA STRUCTURE *3*

- Intracranial Electroencephalography data. 3
- HFO Events 4

INSTALLATION *6*

INTRODUCTION TO THE INTERFACE *7*

- Launching the graphical user interface. 7
- Load data. 8
- Explore data. 10
- Montage. 12

AUTOMATIC HFO DETECTION *14*

- Automated HFO detector. 14
- Implementing a new automatic detector. 17

HFO VISUAL MARKING *19*

- Semi-automatic HFO detection using bandpass-filtered data. 19
- Semi-automatic HFO detection using the spectrogram. 25
- Visual marking of HFOs. 27

RESULTS *30*

TOOLTIPS *31*

- Menu File 31
- Menu Event 31
- Menu Windows 32
- Menu Montage 33
- Menu Spectrogram 34
- Menu Options 35

REFERENCES *37*

SUMMARY

We developed a MATLAB-based graphical user interface, named HFOApp, for visual marking of high frequency oscillations (HFOs) in intracranial electroencephalography (iEEG) recordings. HFOApp is an optimized marking tool with an intuitive interface designed for ease of use, accuracy of markings, and minimization of inter-rater variability. The simple structure of input data and events allow visualization of the results of automatic detection methods within HFOApp in order to aid visual marking. We implemented a simple, interactive, Hilbert detector which significantly reduces the time needed for visual marking of HFOs while reducing inter-rater variability. We hope that the clinicians and researchers who study HFOs will find HFOApp to be an efficient, easy-to-learn, useful tool. Many thanks go to the people who have contributed to this project and to our funding sources.

KEYBOARD SHORTCUTS

‘f’ – Move plots forward by one window length.

‘b’ – Move plots backward by one fourth of a window length.

‘s’ – Mark the start of an event (manual mode).

‘e’ – Mark the stop of an event (manual mode).

‘a’ – Add detected events to the event list box.

‘d’ – Perform multiple-channel events detection.

‘w’ – Turn on/off manual markings mode.

DATA STRUCTURE

Intracranial Electroencephalography data

HFOApp uses *HFOLoadData.m* to load data for analysis. The output of this function is a MATLAB data structure, which is required by the toolbox, and which contains the following fields (**Fig. 1**):

mat, channel x sample.

srate, sampling rate (in Hz).

labels, cell array of channel labels

file, full path to the file.

start_clock, datetime type in the format of "yyyy-MM-dd HH:mm:ss".

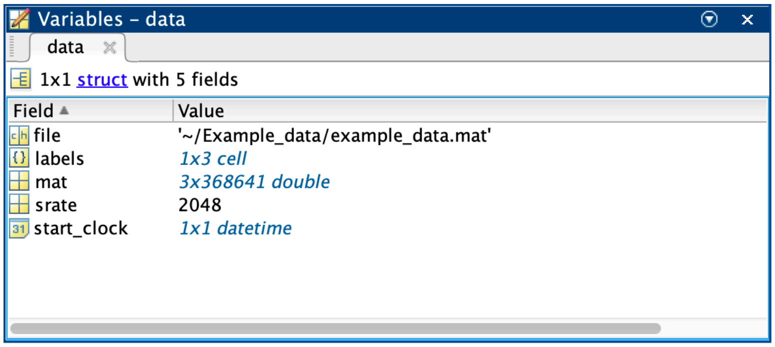

Figure 1. Data structure required by HFOApp.

The data can also include a *time* field, which must be a vector of the same length as that of the data. HFOApp also supports multi-trial/session datasets, in which case the *mat* must be a 3-dimensional matrix (*channel x sample x trial*).

HFO Events

If you want to review and edit HFO events that were detected using a different software or algorithm, you have to organize the events as a structure array containing the following fields (**Fig. 2**):

label, channel name

info, N x 3 cell array of event information, where N is the number of events.

Each row of *info* is a 1 x 3 cell array that stores the location and event type information (**Fig. 2**). The event type must be one or more of "*gamma*", "*ripple*", "*fastripple*", '*ultrafast*", and "*spike*".

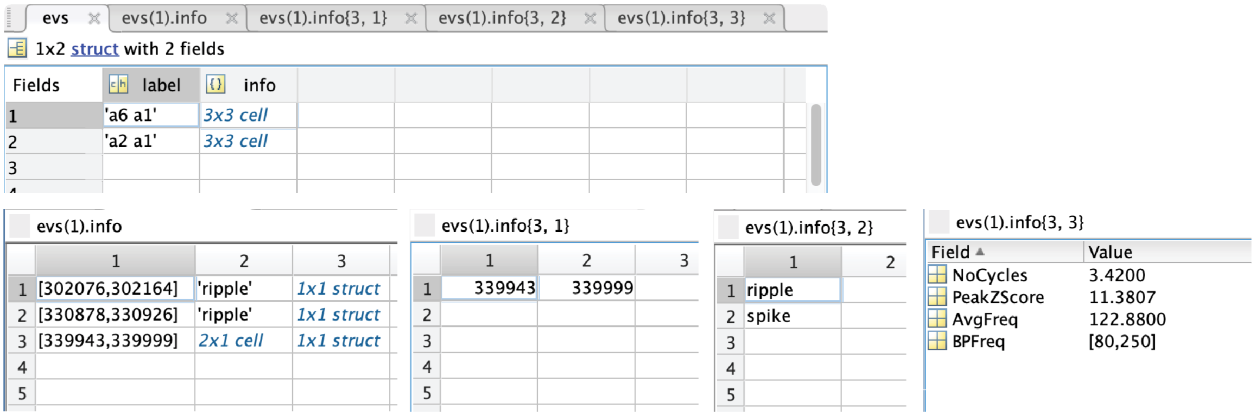

Figure 2. Event structure of single session.

If the input data has multiple trials, the event has to be a cell array, each of which is itself a structure array as described above (**Fig. 3**).

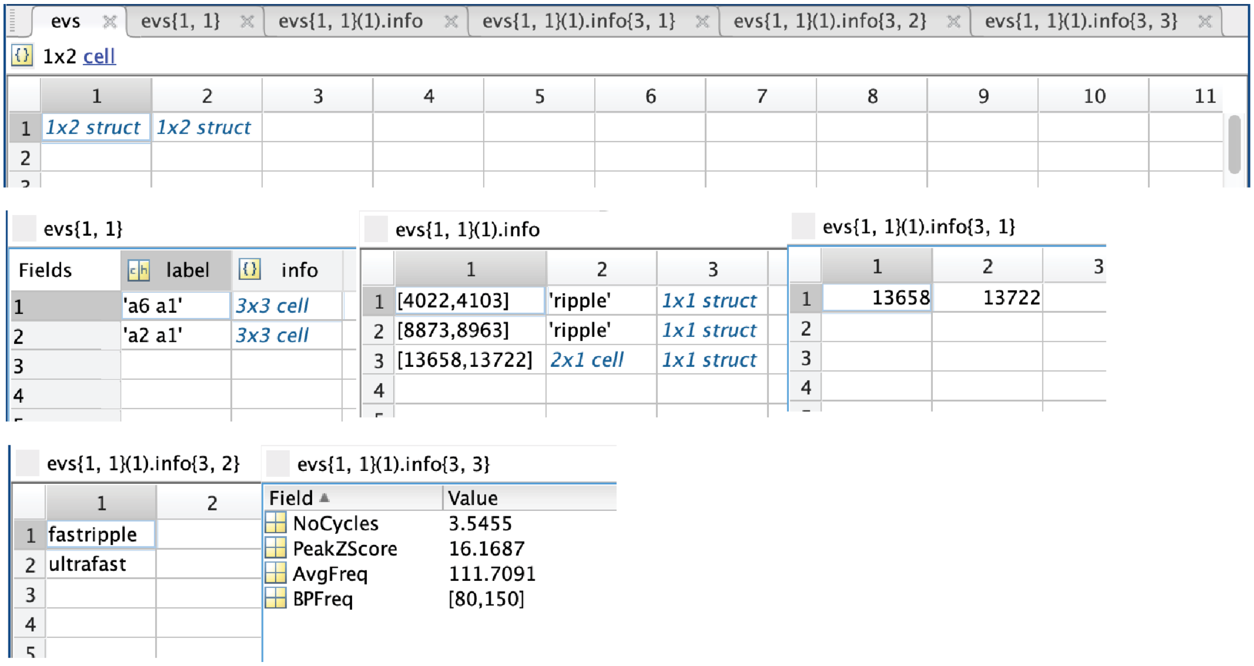

Figure 3. Event structure of multiple sessions.

To find an example of how to organize the data and events, please see *HFOApp_ExampleUsage.m* in the *Functions* folder of HFOApp toolbox.

INSTALLATION

1. Unzip the downloaded toolbox if it's in a compressed format.
2. Add the toolbox folder to the MATLAB working path. You can change the MATLAB working directory to the folder or add it to MATLAB path by editing *startup.m*.

   Go to the MATLAB Command Window and enter:

   edit startup

   Add the following line (replace *'~/Downloads/HFOApp'* with your own directory):

   addpath('~/Downloads/HFOApp');

3. To run HFOApp, go to the Matlab Command Window and enter:

   HFOApp

INTRODUCTION TO THE INTERFACE

Launching the graphical user interface

To launch HFOApp, go to the Matlab Command Window and enter:

HFOApp

A welcome page will show up (**Fig. 4**). You will notice that only the ***File*** menu is enabled. Now you can either go to ***File*** → ***Open*** to load a dataset, or go to ***File*** → ***Quit*** to quit the program.

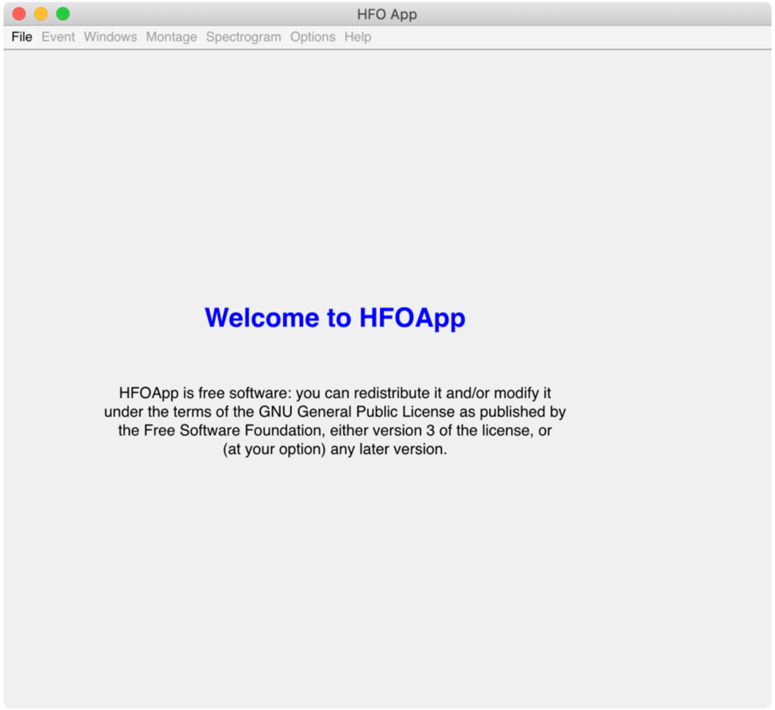

Figure 4. HFOApp launch screen.

Load data

File → ***Open***. A file selection dialog will pop up and ask you to choose the file to load. The toolbox currently supports reading data in Micromed (**.trc*), European Data Format (**.edf*) or Matlab (**.mat*) format. You can edit *HFOLoadData.m* to add support of your own data format.

If the data are loaded successfully, you will first be asked whether to enable auto-saving even before you start marking HFOs (**Fig. 5**). If you select ***Yes***, a dialog window pops up, whichs ask the user to specify a file name to save the results. After doing do, all changes will be saved automatically throughout the marking process. If you select ***No***, you have to remember to save the results after you are done.

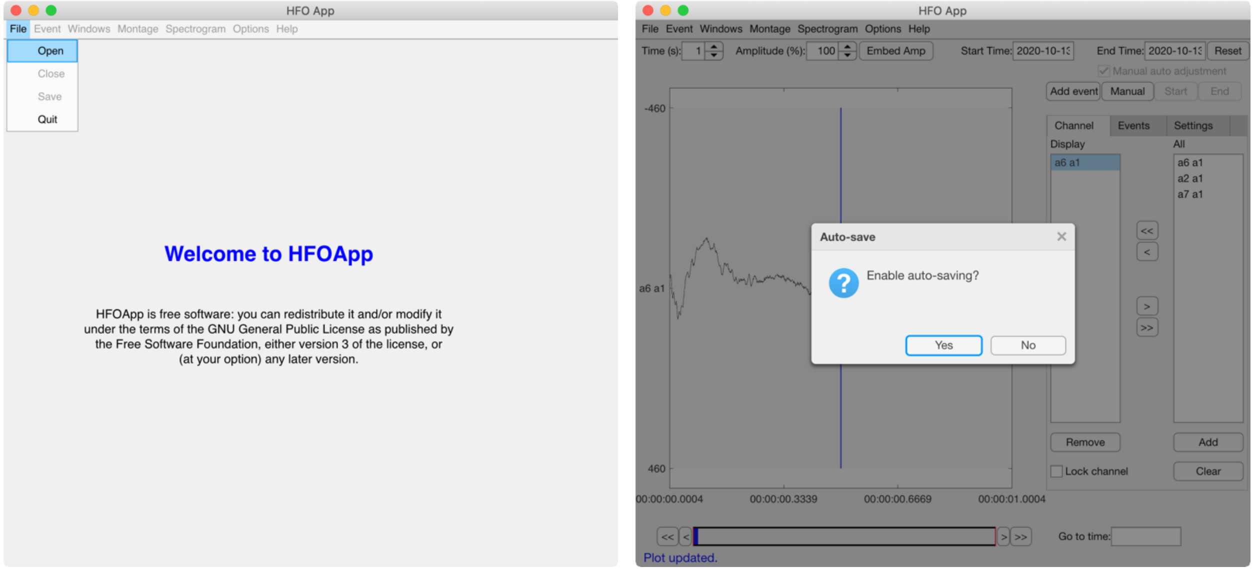

Figure 5. Save results before marking HFOs to enable automatic saving.

Explore data

If the data were loaded successfully, you will see the first channel of the raw data plotted in the window (**Fig. 6**). By default, the first 1 second of the time series is plotted. The length of this time window can be changed by entering a new value into the ***Time (s)*** edit field. To adjust the *y* -axis scale of the plot, enter a new value into ***Amplitude (%)*** edit field.

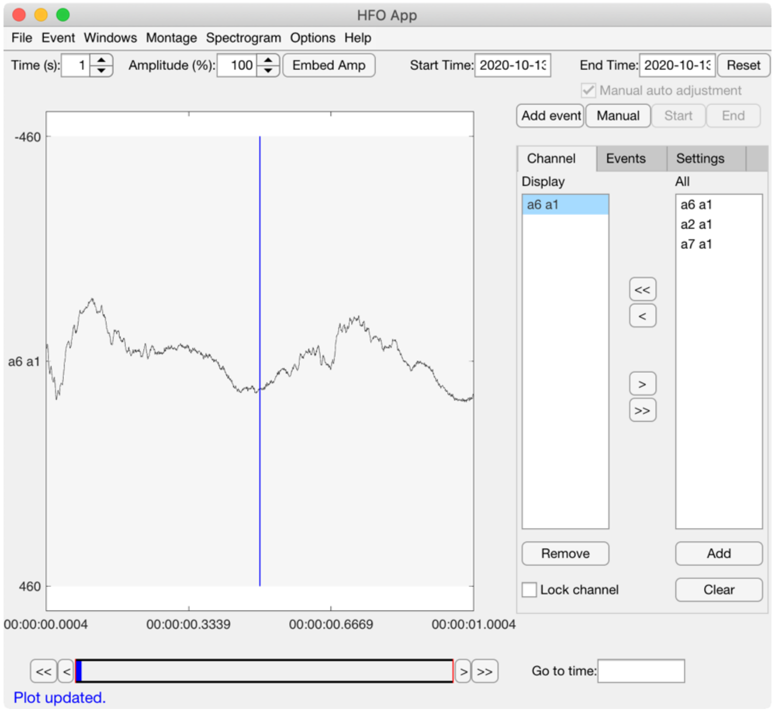

Figure 6. HFOApp startup page after data are loaded.

To the right of the display window, the ***Channel*** tab (**Fig. 6**) is open by default, and you'll see all uploaded data channels listed in the ***All*** box and the current channels being plotted on the display listed in the ***Display*** box. To add channels to ***Display***,

select them in the ***All*** list box and then press the **<** or ***Add*** button. To add all channels to ***Display***, press the **<<** button.

To remove channels from ***Display***, select them in the ***Display*** list box and press the **>** or ***Remove*** button. To remove all channels, press the **>>** button.

To remove channels from loaded data, select the channels in ***All*** list box and press ***Clear.***

To move the plots in the display window backward or forward, press the **<<**/**<** or **>**/**>>** button below the plots (**Fig. 6**). By default, the step size for **<<** and **>>** is the current window length and **<**/**>** is half of the time window length. The step size can be changed in ***Options*** → ***Set preferences*** menu. To move the plots to a specific time, use the ***Go to time*** edit field; you can also move the plots to any rough time point in the data by clicking anywhere in the navigation bar below the main plot—the display will move the time point where you click.

To limit the analysis to a specified time window of interest, change the start and end time in the ***Start Time*** and ***End Time*** edit fields (**Fig. 6**). The time can be in datetime type (*yyyy-MM-dd HH:mm:ss)* or in seconds, which is determined by the clock type (set through ***Options*** → ***Set preferences*** → ***Clock type****).*

Montage

Bipolar reference has been widely used in HFO marking. To re-reference the data using bipolar reference, go to menu ***Montage → Change Montage*** (**Fig. 7**).

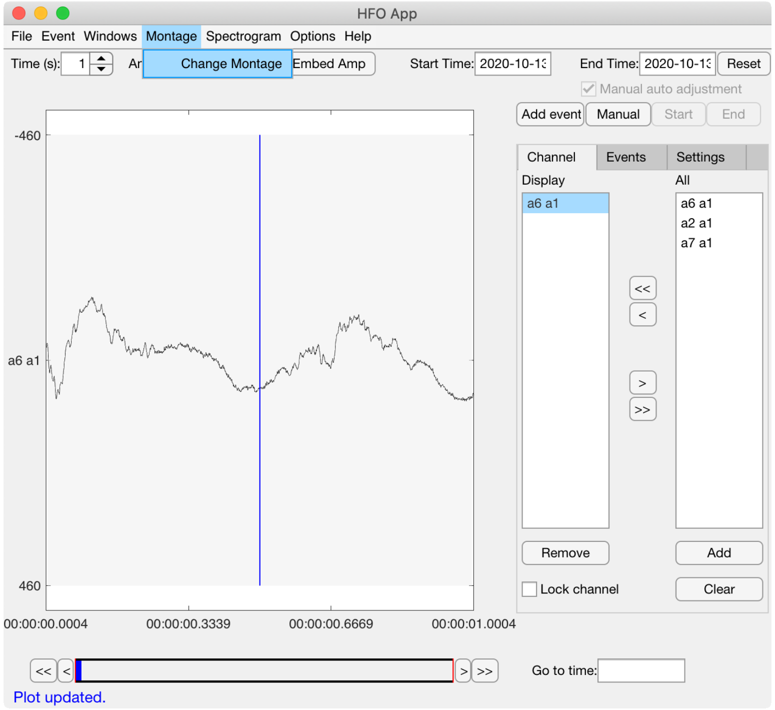

Figure 7. Montage setup in HFOApp.

Select ***Bipolar Reference*** in the pop-up montage setup window (**Fig. 8** left). First, select a channel in the ***All channels*** list box and click the first cell of the table of ***Bipolar reference***. Then click the **>>** button, which will assign the selected channel to the first row and first column of the ***Bipolar reference*** setup table. By default, the selection of current channel will move to the next one in ***All channels*** list box and the cell focus will move to the next in ***Bipolar reference*** (**Fig. 8** right) after the **>>** button is

pressed. To remove a channel pair from ***Bipolar reference***, click on the row number and then press the **<<** button.

To save the re-referenced data into HFOApp format, go to ***File* → *Save***.

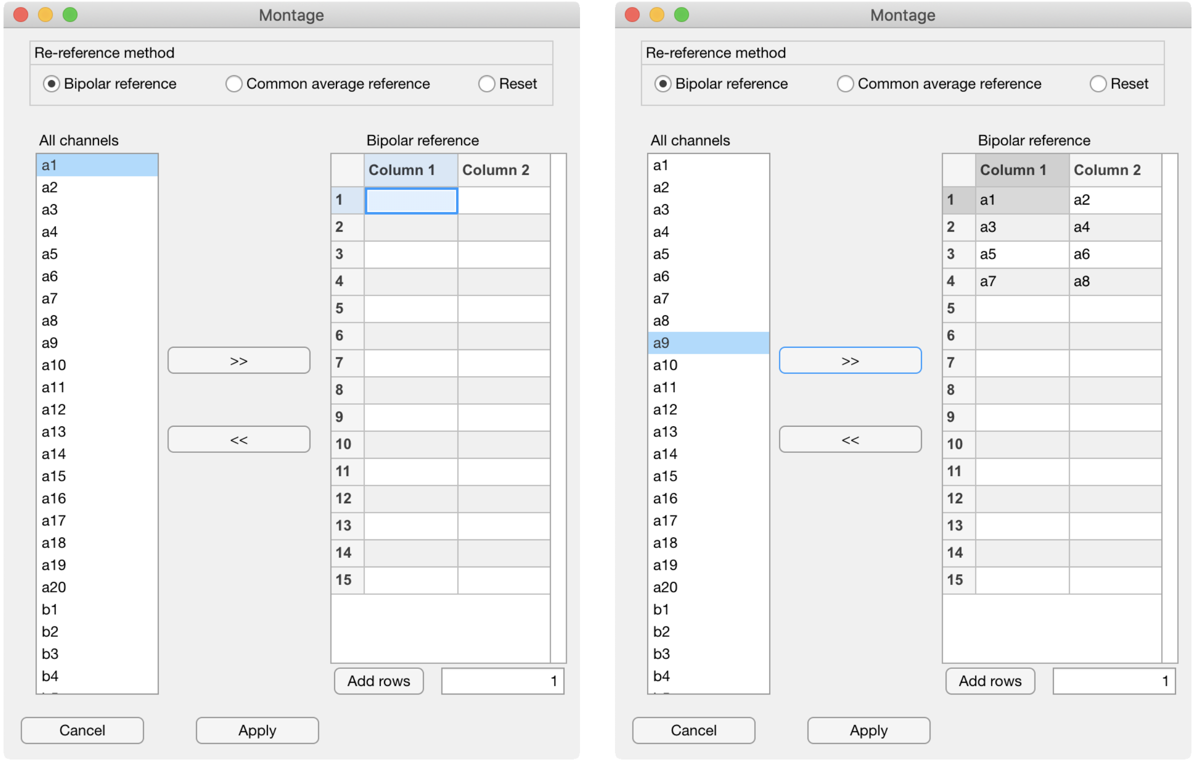

Figure 8. Bipolar re-reference in HFOApp.

AUTOMATIC HFO DETECTION

Automated HFO detector

After the montage is set properly, we can run automated HFO detection on all channels in the ***Display*** list box. Available detection methods include Hilbert Detector **(Crépon et al., 2010; Höller et al., 2018)**, Short Time Energy Detector **(Staba et al., 2002)**, Short Line Length Detector **(Gardner et al., 2007)**, and MNI Detector **(Navarrete et al., 2016; Zelmann et al., 2012)** (**Fig. 9–12)**. Implementation details of these detectors can be found in **Navarrete et al., 2016**. New detection method can be added easily; see the next section.

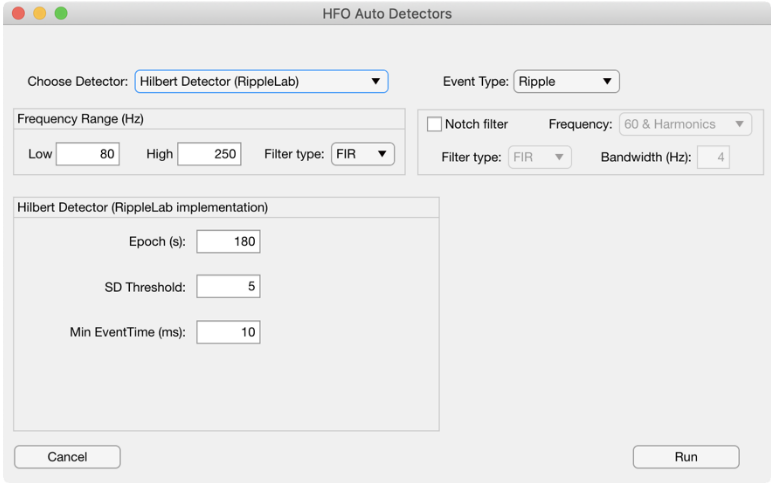

Figure 9. Hilbert detector setup.

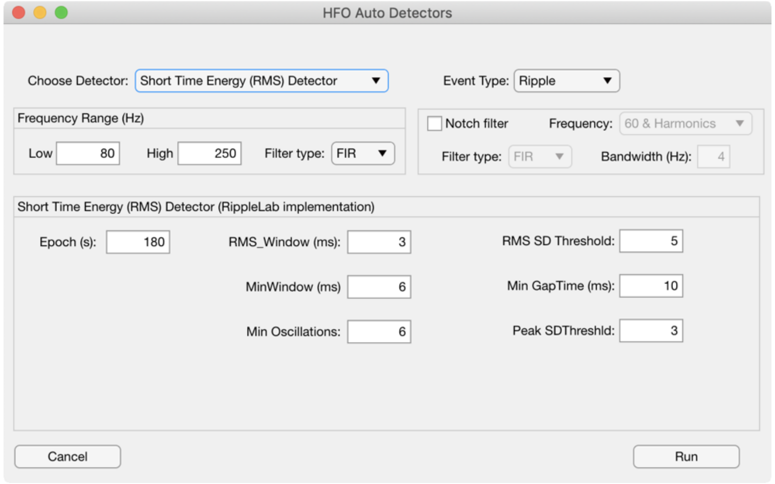

Figure 10. Short time energy detector setup.

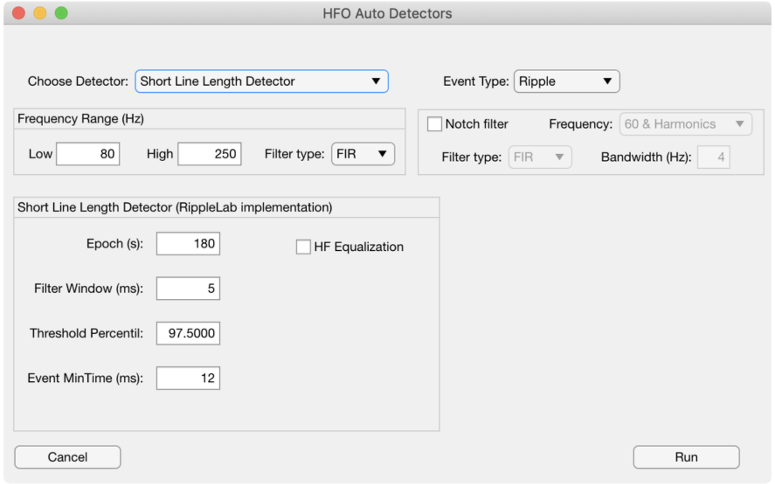

Figure 11. Short line length detector setup.

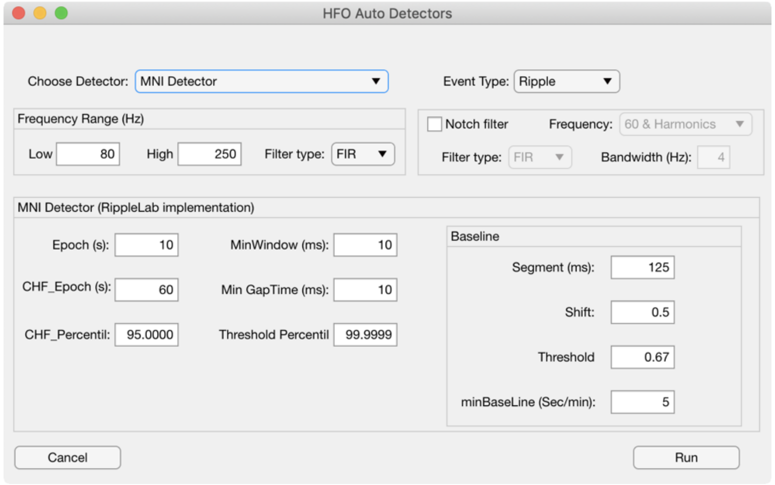

Figure 12. MNI detector setup.

Implementing a new automatic detector

To implement a new detector in HFOApp, the user only needs to edit two files: *HFOAutoDetect.m* (implementation of the algorithm) and *ConfigAutoDetector.mlapp* (GUI configuration).

HFOAutoDetect was designed to be independent from the GUI itself. The *HFOAutoDetect* takes data matrix, sampling rate, channel labels and a configuration data structure as input. The output is organized as a structure array of detected events.

```
function evs = HFOAutoDetect( mat, srate, labels, cfg)
```

To add a new detector in HFOApp GUI, open *ConfigAutoDetector.mlapp* and create a panel like ***MNI Detector*** in **Fig. 12**. Then add the name of detector to the list of detectors (***Choose detector*** dropdown box in **Fig. 12**) and edit the callback of the dropdown box (function *DetectorDropDownValueChanged*), which controls which configuration to show when the detection method changes. Finally, go to function *RunButtonPushed* and find the section that contains the lines below:

```
%% Add customized detector here
    detec_method = app.DetectorDropDown.Value;
    if strcmp( detec_method, 'Hilbert Detector (HFOApp)')
        cfg.method = 'hil';
        cfg.epoch = app.HilEpochsEditField.Value;
        cfg.nocycle_thresh = app.HilThreshNumberCyclesEditField.Value;
      cfg.z_thresh = app.HilThreshOnsetEditField.Value;
      cfg.peak_thresh = app.HilThreshInclusionEditField.Value;
```

All you need to do is to add an *elseif* statement to setup the *cfg* properly so that it can be accepted by *HFOAutoDetect.m*.

HFO VISUAL MARKING

Semi-automatic HFO detection using bandpass-filtered data

In HFOApp, it is easy to specify the channel or channels for analysis. You can set the channel to analyze by left-mouse clicking the desired plot in the display window or by clicking the channel label in the ***Display*** list box. If the channel to analyze was set, the background of the corresponding plot will become gray. The color of both of the background and time series can be changed in ***Options*** → ***Set preferences.*** To lock marking to the current channel, check the ***Lock channel*** check box. Locking the channel will prevent marking on any other channel until the ***Lock channel*** check box is unchecked.

To perform semi-automated event detection using the Hilbert detector **(Crépon et al., 2010)**, a bandpass-filter window must be opened. Go to menu ***Windows*** → ***Add bandpass-filter window*** (**Fig. 13** left). A new window will pop up and the user has to enter the parameters for bandpass filtering (**Fig. 13** right). The bandpass-filtered data can be plotted in a separate window (check the ***Open a new window*** check box), or it can be plotted below the raw time series in the main window (uncheck the ***Open a new window*** check box).

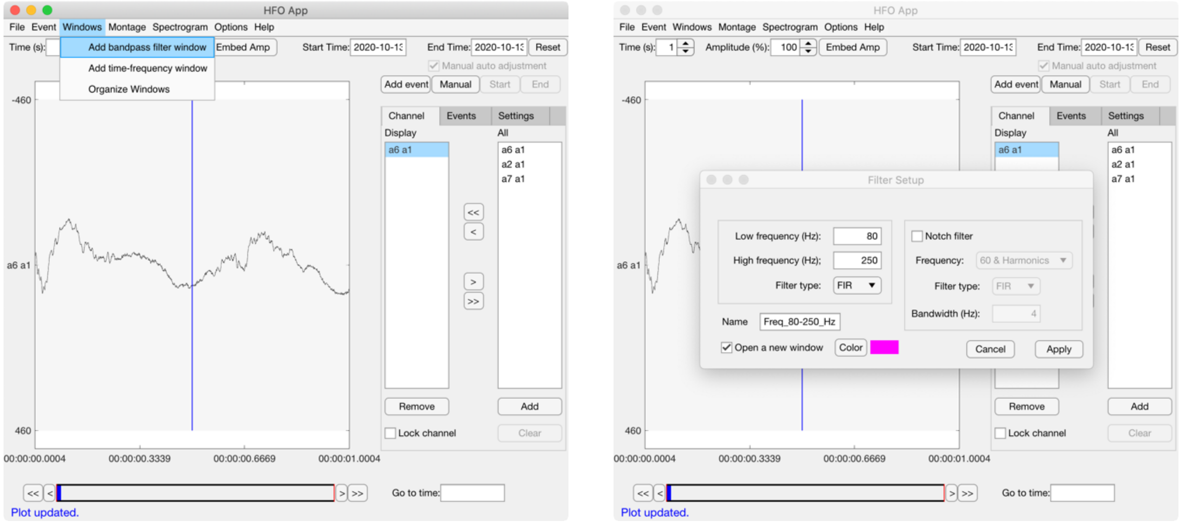

Figure 13. Bandpass-filter window setup.

HFOApp supports multiple bandpass-filter windows. Please make sure to use a different name for each window. To organize the bandpass-filter windows, go to menu ***Windows*** → ***Organize Windows*** **(Fig. 14).**

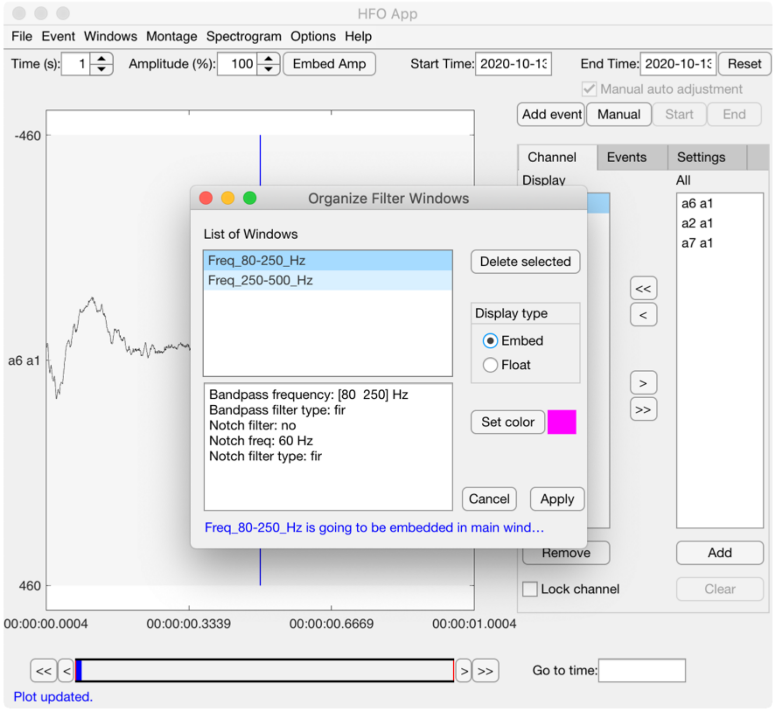

Figure 14. Organize bandpass-filter windows.

To add a spectrogram window for the current channel, go to the ***Windows*** menu and click ***Add time-frequency window*** (**Fig. 14**). A new spectrogram window, with the label of the current channel as its title, opens up after a few seconds, depending on the length of the data. You can change the default settings for the spectrogram calculation in menu ***Spectrogram*** → ***Configure***.

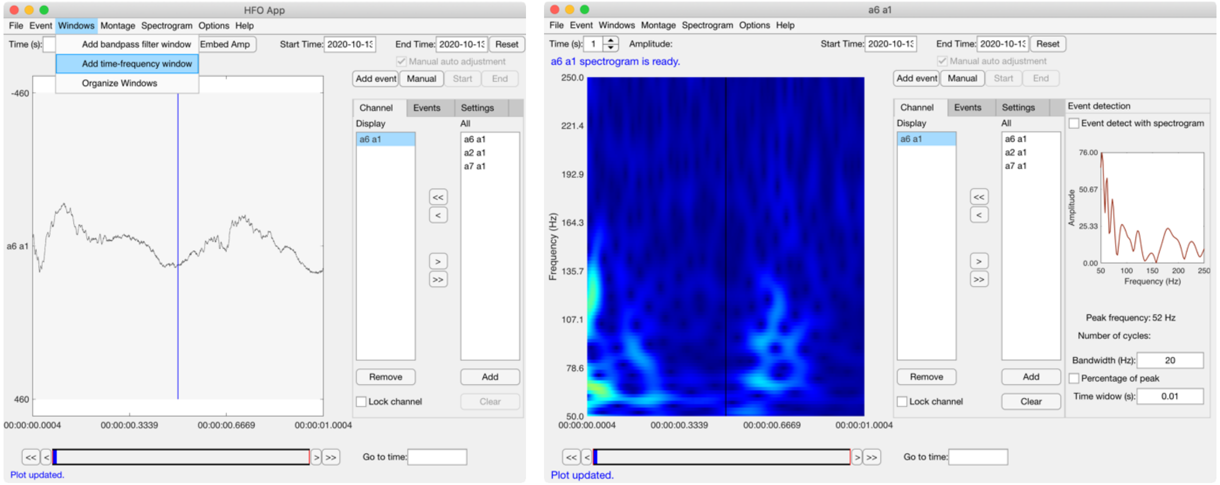

Figure 8. Open a spectrogram window.

You can now return to the bandpass-filter window to start HFO detection. Note, if you are using only one bandpass-filter window, it won't be matter whether you work on the main window or bandpass-filter window. However, if you are using multiple bandpass-filter windows, you have to click on the plot in the specific window that you want to analyze for event detection.

To start semi-automated event detection, left click on bandpass-filtered time series plot. The background of selected channel will become gray. A summary information of the click is displayed at the top of the display window. If an event is detected, a red dashed

rectangle appears surrounding that event (**Fig. 15**). To add the detected event to results, go to ***Event*** tab, select the event type and press the ***Add Event*** button. The event will appear in the event list box. Parameters for threshold and event detection can be changed in the ***Settings*** tab.

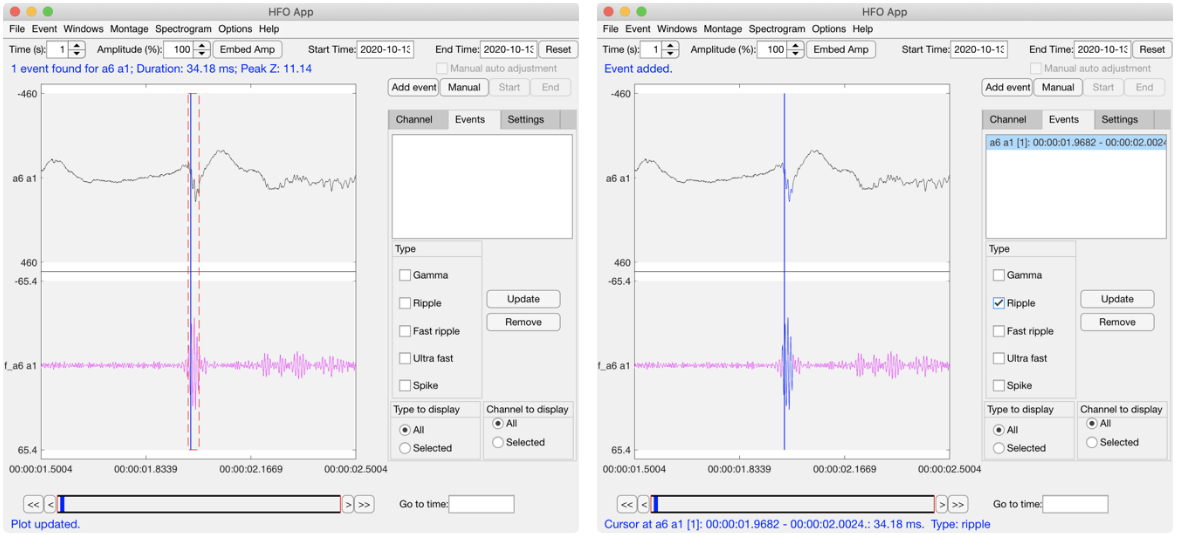

Figure 15. An example of detected high frequency oscillations event.

To update the ***Type*** of an existing event, select the event in the ***Events*** list box. Its current information will be shown at the bottom of the window. You can change the event type by selecting a new event type in the ***Type*** box, and clicking the ***Update*** button. If you want to remove the event from the ***Events*** list, press ***Remove*** button.

The algorithm searches for events within a specific range of the cursor location. If you increase this range, more events could be detected. To change default values, go to ***Options*** → ***Set Preferences*** and enter new values in ***Search window (s)*** edit field (**Fig. 16**).

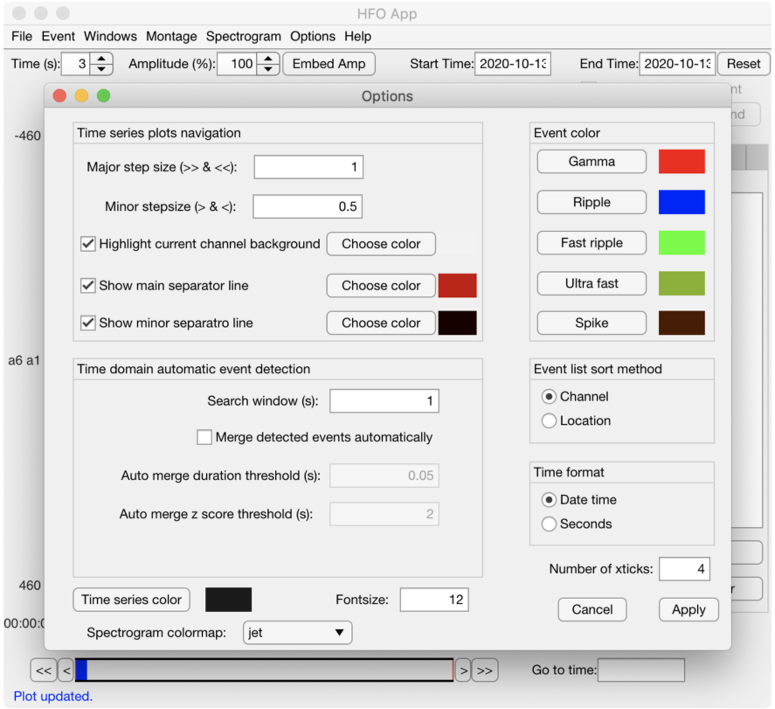

Figure 16. Change default event search options in the Time domain automatic event detection panel.

By default, HFOApp searches for events within a 1 s time window centered at the cursor location for the selected channel. To enable auto detection on all channels in the ***Display*** list box, first go to the ***Settings*** tab and check the ***Enable multiple channel detection*** check box (**Fig. 17**). Then, increase the ***Search window*** value (**Fig. 16**) to make it large enough for multiple-events detection. Now, click on any channel and press ***d*** on the keyboard and all detected events on all displayed channels will be outlined. Right-mouse click within the red rectangle to deselect any single identified event.

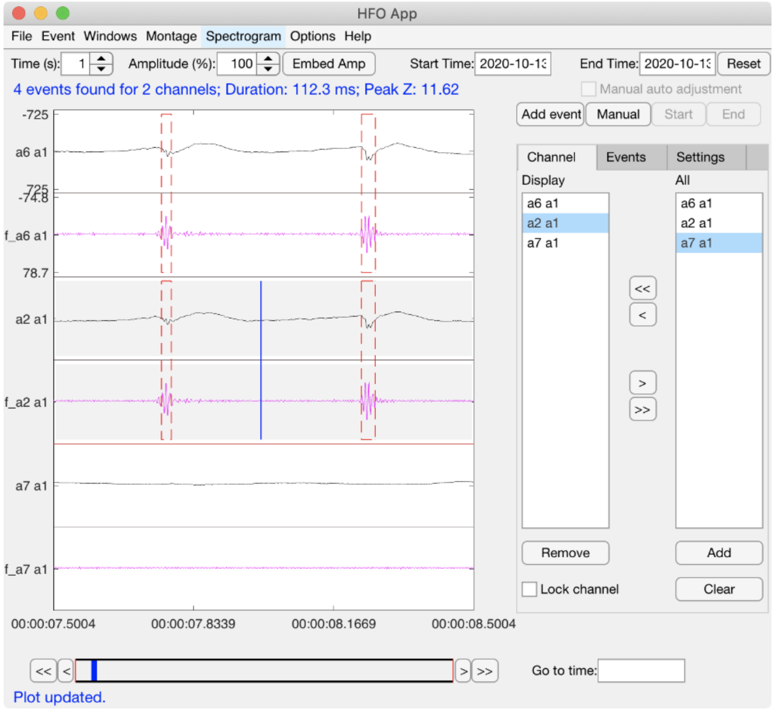

Figure 17. Event detection on multiple channels.

The user can also enable automatic merging of two adjacent events if: 1) the distance between the two events is less than a threshold, and 2) all z score values between these events are greater than a specified threshold (**Fig. 16**).

Semi-automatic HFO detection using the spectrogram

To detect events using the spectrogram, go to the spectrogram window and check the ***Event detect with spectrogram*** check box (**Fig. 18**). Please note, multiple event detection is automatically disabled in the spectrogram detection mode. To start the detection, click within the spectrogram on any hotspot that you're interested in and a vertical black line will appear at that location. If an event is detected, a red dashed rectangle will appear surrounding the event. This method will check the spectrogram plot as a function of the frequency at the cursor location within the spectrogram window: clicking in the spectrogram will prompt the autodetection to find the peak frequency of the average over a small-time window centered at the cursor location (***Time window (s)*** in the ***Event detection*** panel, **Fig. 18**). Then, the raw time-series is bandpass filtered at a frequency band centered at the peak frequency (the bandwidth is user-specified in ***Bandwidth (Hz)*** edit field). Finally, the same algorithm used in the bandpass-filter window event detection is used within this bandpass-filtered window to detect events.

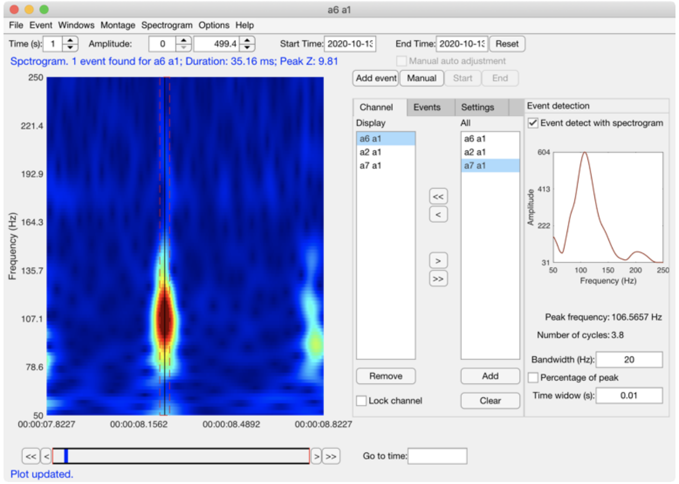

Figure 18. Event detection using the spectrogram.

The bandwidth can also be determined based on the peak amplitude of the spectrogram by selecting the ***Percentage of peak*** check box: The frequencies at which the amplitude drops to that percentage of the peak will be defined as the low- and high-cutoff frequencies of the bandpass filter.

Visual marking of HFOs

So far, we have introduced semi-automatic detection. In this section, we will introduce the manual marking mode.

To enable the manual marking mode, left mouse click on the ***Manual*** button (**Fig. 19**). This will enable the manual marking options: the ***Start*** and ***End*** buttons are enabled, and the ***Manual auto adjustment*** check box is enabled. To proceed without the ***Manual auto adjustment*** option, leave the ***Manual auto adjustment*** checkbox unchecked. In this mode, the user can scroll through the time-series looking for events visually. When an event is found, left mouse click on the time series or the spectrogram plot, and then press ***Start*** and ***End*** buttons to mark the start and end point of an event; an event between the start and end points will be highlighted on the time-series (**Fig. 19**) between the selected points.

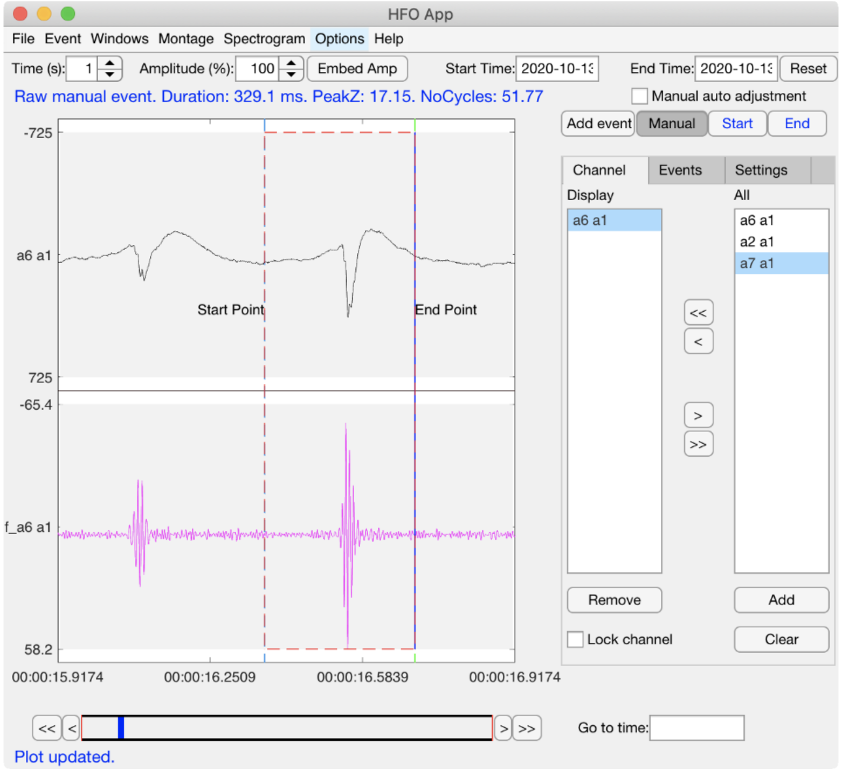

Figure 19. Manual event without automatic adjustment.

If ***Manual auto adjustment*** is checked, the start and end points will be automatically adjusted after they are selected based on the automatic event detect parameters set by the user (see ***Settings*** tab). If no cluster within this manual event selection survive the thresholds, the dashed red rectangle will not change; if the events do survive the thresholds the onset and offset of the red rectangle will be adjusted (**Fig. 20**). Please note, only one event on the current channel can be marked at a time in this mode. In addition, at least one band pass filter window must be open for the ***Manual auto adjustment*** to work. If more than one bandpass-filter window is open, the last active window will be used.

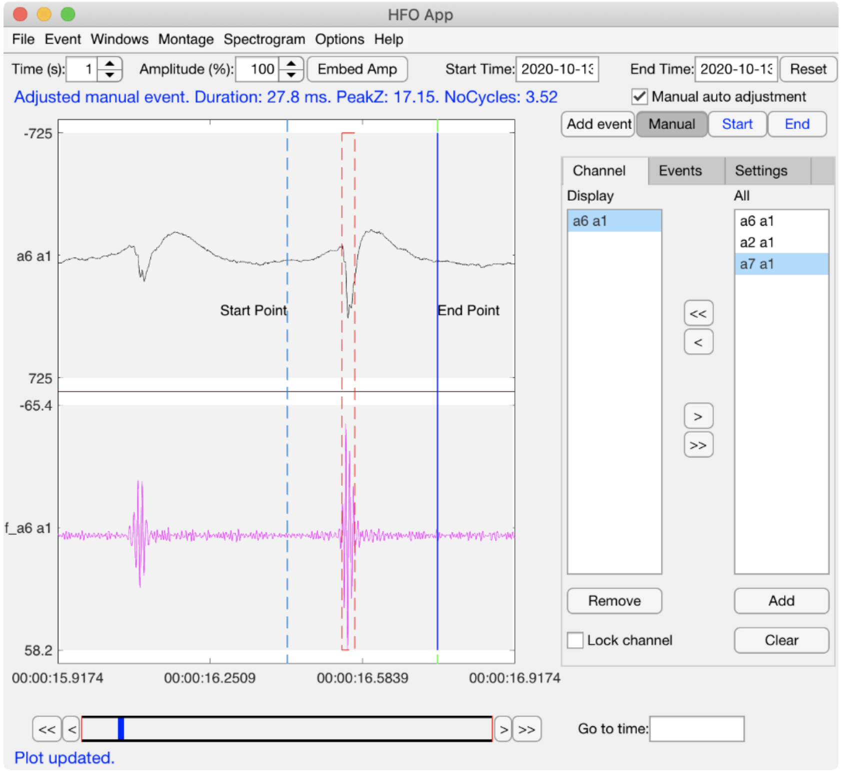

Figure 20. Mark event with automatic adjustment.

In ***Manual auto adjustment*** mode, the manually marked events may be divided into two or more events. These events can be merged automatically if: 1) the distance between the two events is less than a threshold, and 2) all z score values between these events are greater than a specified threshold (**Fig. 16**), which is similar to the automatic merge in auto detection that we introduced above.

For the visual marking, the user can display raw and bandpass-filtered data next to each other in the same window or in different windows. To switch to a channel to mark HFOs, the user just simply click on the channel, either on the time-series or on the channel label in the **Display** list box, and the corresponding channel will be highlighted.

RESULTS

The detected events will be saved in a comma delimited text file. Each row indicates one detected event along with its information, including location and event type (**Fig. 21**). HFOApp also generates a summary file that reports the statistics of high frequency oscillations events for each channel (**Fig. 22**).

HFOEvents_example_data_st.txt

```
File created by HFOApp. DO NOT EDIT.
Number of header lines:  11
Number of trials: 1
Data file: ~/Desktop/Utilities/HFO/HFOAPP/example_data/example_data_st.mat
Time: 2020-10-13 00:00:00 - 2020-10-13 00:03:00
Total number of channels: 3
Length of data: 180.0005 s
Sampling rate: 2048 Hz
Onset threshold: 3; Inclusion threshold: 5; Number of cycles: 3.
----------------------------------------------------------------------------------------------------
Trial, Channel, EventLocation(sample),  EventType, AverageFreq(Hz), PeakZscore, NoCycles,       BPFreq
    1,   a6 a1,         302076  302164,     ripple,          110.70,       9.53,     4.81,   80.0  250.0
    1,   a6 a1,         330878  330926,     ripple,          128.00,      12.38,     3.06,   80.0  250.0
    1,   a6 a1,         339943  339999,     ripple,          122.88,      11.38,     3.42,   80.0  250.0
    1,   a2 a1,         300952  301005,     ripple,          117.03,       6.40,     3.09,   80.0  250.0
    1,   a2 a1,         302075  302145,     ripple,          113.78,       9.38,     3.94,   80.0  250.0
    1,   a2 a1,         330876  330926,     ripple,          124.12,      12.09,     3.09,   80.0  250.0
```

Figure 21. Example of HFOApp created high frequency oscillations event file.

HFOEvents_example_data_st_summary_19-Nov-2020-16-08-52.txt

```
File created by HFOApp. DO NOT EDIT.
Number of header lines:  11
Number of trials: 1
Data file: ~/Desktop/Utilities/HFO/HFOAPP/example_data/example_data_st.mat
Time: 2020-10-13 00:00:00 - 2020-10-13 00:03:00
Total number of channels: 3
Length of data: 180.0005 s
Sampling rate: 2048 Hz
Onset threshold: 3; Inclusion threshold: 5; Number of cycles: 3.
----------------------------------------------------------------------------------------------------
NoTrial, Channel, GammaDur(s), RippleDur(s), FastrippleDur(s), UltrafastDur(s), SpikeDur(s), GammaNo, RippleNo, FastrippleNo, UltrafastNo, SpikeNo
      1,   a6 a1,       0.000,        0.094,            0.000,           0.000,       0.000,       0,        3,            0,           0,       0
      1,   a2 a1,       0.000,        0.084,            0.000,           0.000,       0.000,       0,        3,            0,           0,       0
```

Figure 22. Example of HFOApp created high frequency oscillations event summary file.

TOOLTIPS

Menu File

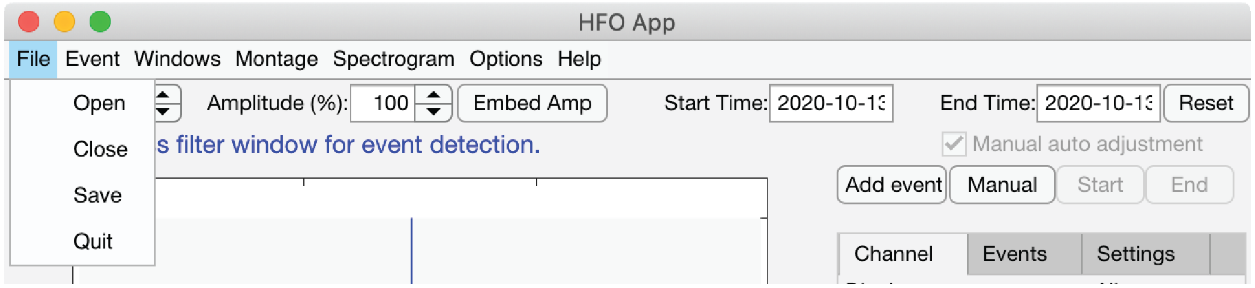

Open	Opens a dialog window to select file.
Close	Close current file.
Save	Save current file in HFOApp format.
Quit	Quit HFOApp. All windows will be closed.

Menu Event

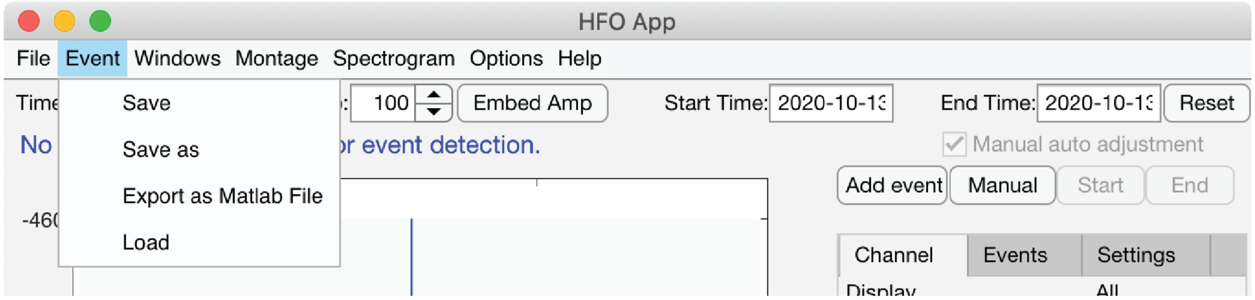

Save	Save HFO events to current result file.
Save as	Save HFO events to a new file.
Export as Matlab File	Export HFO events as Matlab data structure.
Load	Load existing HFO events from *.txt or *.mat file.

Menu Windows

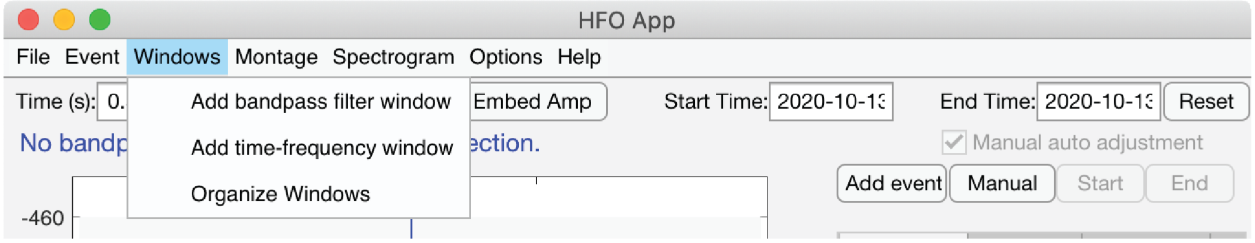

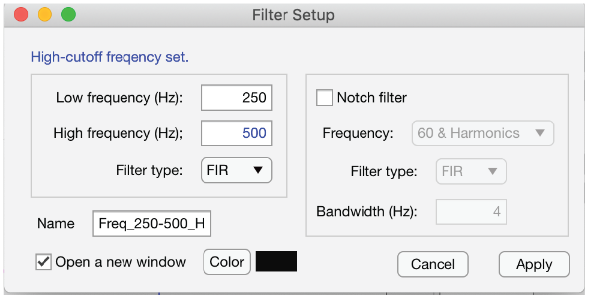

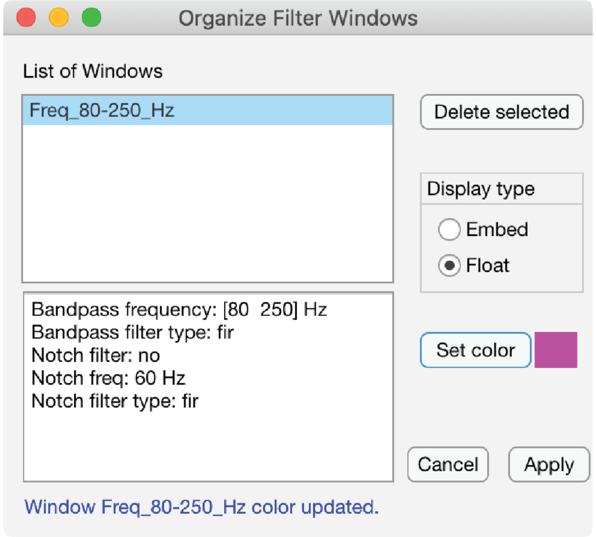

Add bandpass filter window	Open a bandpass configure dialog window.
Add time-frequency window	Open a spectrogram window for the current channel.
Organize Windows	Re-organize the bandpass filter windows.
Filter setup left panel	
Low Frequency (Hz)	Low cutoff of the bandpass frequency.
High Frequency (Hz)	High cutoff of the bandpass frequency.
Filter type	Filter type of the bandpass filter (FIR/IIR).
Name	Name of the bandpass filter window. Please do not use special characters.
Open a new window	Open a new bandpass filter window, or embed the bandpass filtered data into the main window.
Filter setup right panel	
Notch filter	Check this box to apply a notch filter.
Frequency	Power line noise frequency.
Filter type	Filter type of the notch filter (FIR/IIR).
Bandwidth (Hz)	Bandwidth of the notch filter.
Cancel	Cancel adding bandpass filtered data.
Apply	Apply the setup.
Organize Filter Windows	
List of Windows	A list of all bandpass filter windows. The detail of the setup is given in the text area below the list.
Delete selected	The selected bandpass filter data will be deleted after Apply button is pressed.

Display type	Embed the bandpass filtered data into main window (Embed), or display the bandpass filtered data in a separate window (Float).
Set Color	Change the color of the filtered time series.
Cancel	Cancel all changes.
Apply	Apply all changes.

Menu Montage

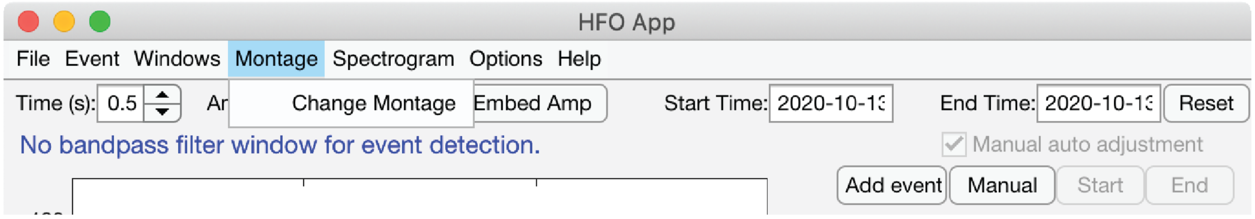

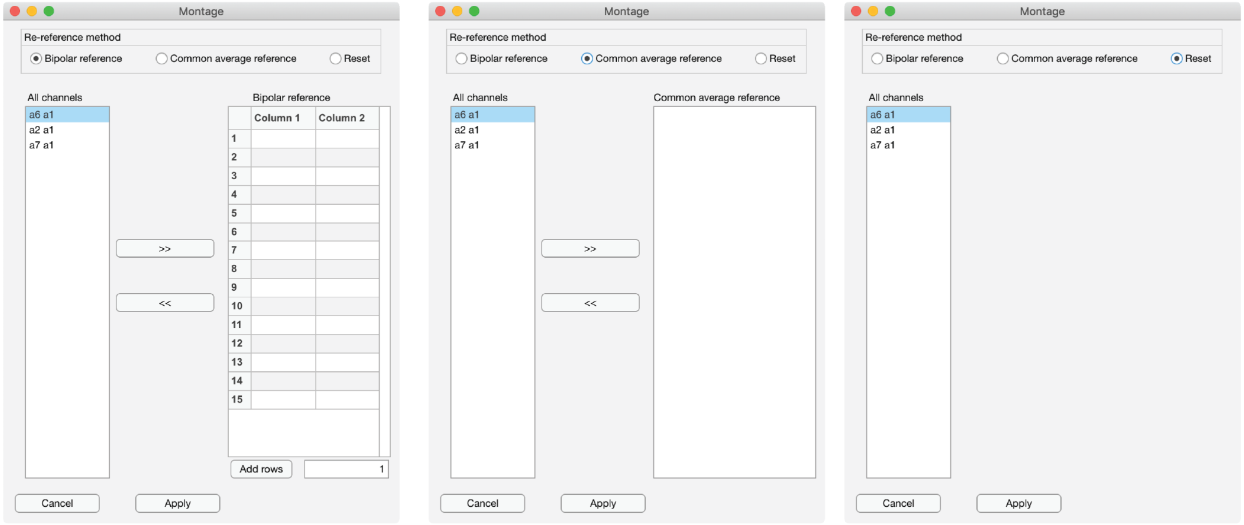

Change Montage	Open Montage configuration window.
Montage	
Re-reference method	Select which re-reference method to use. It can either be Bipolar reference or Common average reference. Reset will revoke any previous re-referencing.
All Channels	A list of all channels of the current file.
>>	Assign selected channel in All channels to a table cell (for Bipolar reference) or list (for Common average reference) on the right.
<<	Remove selected channel in the table/list on the right.
Cancel	Cancel all changes.
Apply	Apply all changes. All bandpass filter and spectrogram windows will be closed.

Menu Spectrogram

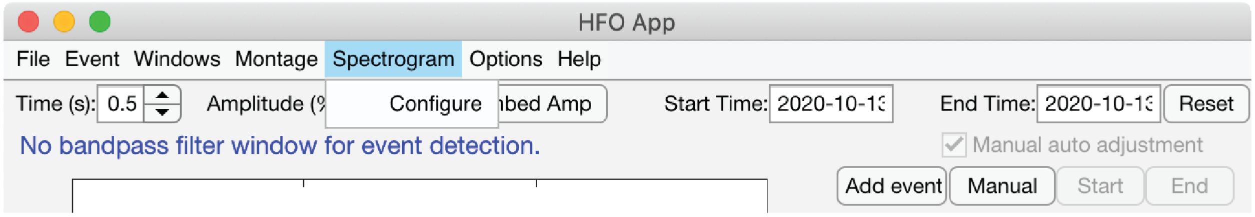

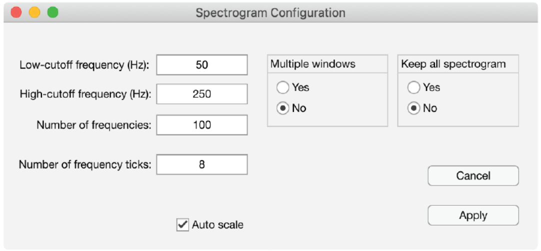

Configure	Configure time-frequency decomposition parameters.
Spectrogram Configuration	
Low-cutoff frequency (Hz)	Low limit frequency of the time-frequency decomposition.
High-cutoff frequency (Hz)	High limit frequency of the time-frequency decomposition.
Number of frequencies	This determines the frequency resolution.
Multiple windows	Whether to allow to open multiple spectrogram windows. If Yes, Keep all spectrogram will be set to Yes automatically.
Keep all spectrogram	Keep the time-frequency results in memory. This will make it faster to re-open a spectrogram window for a channel whose time-frequency result had already been calculated.
Cancel	Cancel all changes.
Apply	Apply all changes.

Menu Options

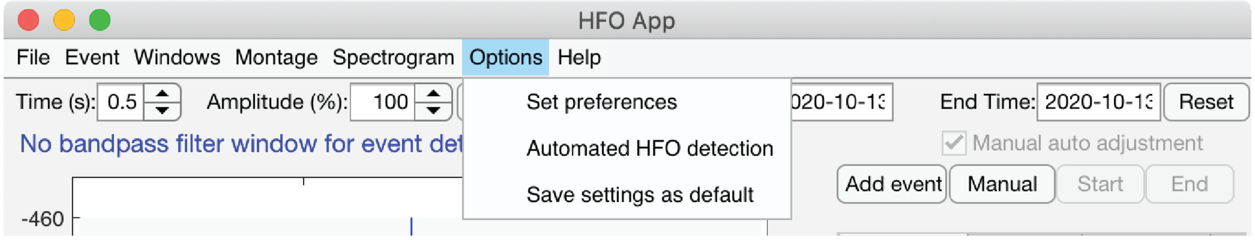

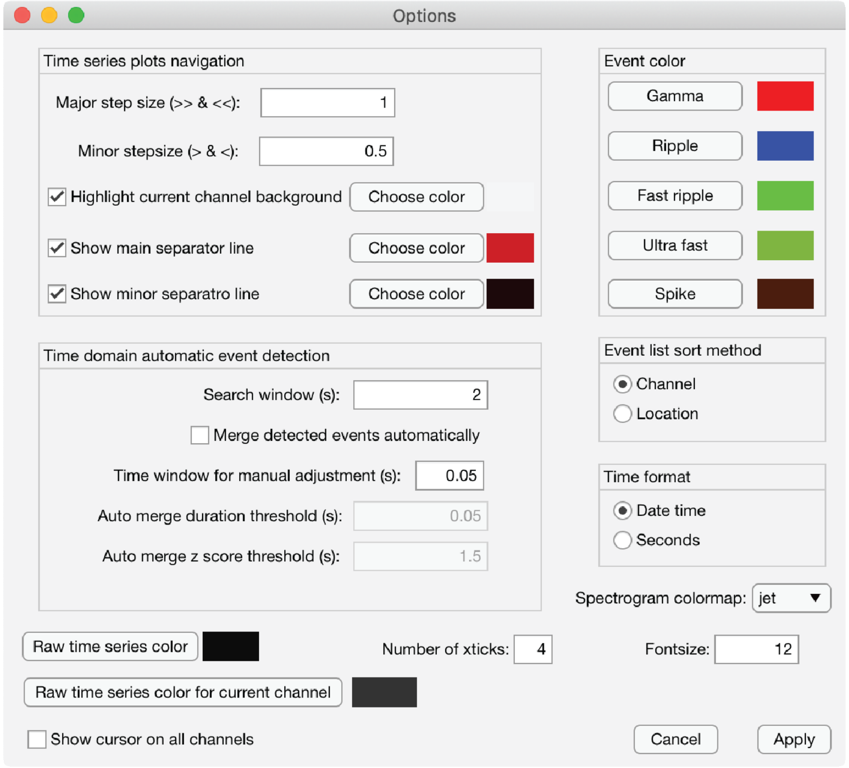

Set preference	Miscellaneous options.
Automated HFO detection	Run automatic HFO detection
Save settings as default	Save all current settings as default.
Help	Information of current file.
Time series plots navigation	
Major step size (>>&<<)	The amount that the plots move when the buttons >> or <<, or "f" on keyboard is pressed.
Minor step size (> & <)	The amount that the plots move when the buttons > or <, or "b" on keyboard is pressed. Both Major and Minor step sizes is relative to the window size, it must be a value between 0 and 1.
Highlight current channel	Whether add a background to current channel or not. The background color can be changed

	using Choose color.
Show main separator line	Separator between channels.
Show minor separator line	Separator between bandpass filter time series in the Main window.
Time domain automatic event detection	
Search window (s)	Window length (centered at the cursor) for HFO event detection.<br>This has to be large enough to make sure the event search convers the current time window. Typical it can be two times of the time window (Time (s)).
Merge detected events automatically	Check this option to enable automatic merge of detected events when the following two criteria are met.
1). Auto merge duration threshold (s)	The distance between events must be smaller than this value.
2). Auto merge z score threshold (s)	The z score of all time points between events must be smaller than this value.
Time window for manual adjustment (s)	Length of the time window which the adjustment of manual events will be limited to.
Raw time series color	Color of the time series in the Main window.
Raw time series color for current channel	Color of the time series of the current channel.
Show cursor on all channels	Check this option to see the cursor across all channels.
Number of ticks	Number of xticks.
Event color	Change the color of each event.<br>If one event has multiple types, the color of the last event type (in the order it appears in Event color) will be used.
Fontsize	Font size of the objects in the windows.
Event list sort method	Sort the events either by Channel or Location of the events.
Time format	Show time as datetime or in seconds.
Spectrogram colormap	Change colormap of the spectrogram.
Cancel	Cancel all changes.
Apply	Apply all changes.

REFERENCES

Crépon, B., Navarro, V., Hasboun, D., Clemenceau, S., Martinerie, J., Baulac, M., Adam, C., and Le Van Quyen, M. (2010). Mapping interictal oscillations greater than 200 Hz recorded with intracranial macroelectrodes in human epilepsy. Brain *133*, 33–45.

Gardner, A.B., Worrell, G.A., Marsh, E., Dlugos, D., and Litt, B. (2007). Human and automated detection of high-frequency oscillations in clinical intracranial EEG recordings. Clin. Neurophysiol. *118*, 1134–1143.

Höller, P., Trinka, E., and Höller, Y. (2018). High-frequency oscillations in the scalp electroencephalogram: mission impossible without computational intelligence. Comput. Intell. Neurosci. *2018*, 1–9.

Navarrete, M., Alvarado-Rojas, C., Le Van Quyen, M., and Valderrama, M. (2016). RIPPLELAB: A comprehensive application for the detection, analysis and classification of high frequency oscillations in electroencephalographic signals. PLoS One *11*.

Oostenveld, R., Fries, P., Maris, E., and Schoffelen, J.-M. (2011). FieldTrip: Open source software for advanced analysis of MEG, EEG, and invasive electrophysiological data. Comput. Intell. Neurosci. *2011*, 156869.

Staba, R.J., Wilson, C.L., Bragin, A., Fried, I., and Engel, J. (2002). Quantitative analysis of high-frequency oscillations (80-500 Hz) recorded in human epileptic hippocampus and entorhinal cortex. J. Neurophysiol. *88*, 1743–1752.

Zelmann, R., Mari, F., Jacobs, J., Zijlmans, M., Dubeau, F., and Gotman, J. (2012). A comparison between detectors of high frequency oscillations. Clin. Neurophysiol. *123*, 106–116.
